# Assessment of genetic susceptibility in patients with oral squamous cell carcinoma: a systematic review and meta-analysis

**DOI:** 10.1038/s43856-026-01398-9

**Published:** 2026-03-20

**Authors:** Kiran Jadhav, Deepak G. S. Pateel, Sonal Grover, Shilpa Gunjal, Srikant Natarajan, Sumanth K. Nagraj

**Affiliations:** 1 Department of Oral Pathology and Microbiology, Vasantdada Patil Dental College and Hospital, Maharashtra University of Health Sciences, Sangli, Maharashtra India; 2 Department of Oral and Maxillofacial Sciences, Faculty of Dentistry, MAHSA University, Selangor, Malaysia; 3 Department of Oral and Maxillofacial Pathology, Christian Dental College, CMC, Ludhiana, Punjab India; 4 Division of Clinical Oral Health Sciences, School of Dentistry, IMU University, Kuala Lumpur, Malaysia; 5 Department of Oral Pathology and Microbiology, Manipal College of Dental Sciences Mangalore, Manipal Academy of Higher Education, Manipal, India; 6 Peninsula Dental School, University of Plymouth, Plymouth, UK

**Keywords:** Oral cancer, Cancer genetics

## Abstract

**Background:**

Oral squamous cell carcinoma (OSCC) is associated with many risk factors but not all individuals who are exposed to the risk factors develop OSCC. This aspect warrants the need to understand the genetic susceptibility in presence of risk factors.

**Methods:**

The study protocol has been registered with PROSPERO (CRD42023422519). The present study included the English-language literature published from January 2000 to April 2024. The articles were searched using keywords via Ovid platform through various databases. The odds ratio was considered as standard measure of outcome. Meta-analysis was carried out using random effects model with RevMan 5.4, establishing a 95% confidence interval and a significance threshold of *p* ≤ 0.05.

**Results:**

Taiwan, China, India, and USA share the major bulk of studies. The subgroup analysis shows polymorphism in P53 gene in presence of smoking (*Z* = 2.15, *p* = 0.02), alcohol (*Z* = 2.38, *p* = 0.02) and mixed habit (tobacco + alcohol) (*Z* = 3.28, *p* ≤ 0.001) and CASP 8 (*Z* = 5.38, *p* ≤ 0.0001) in presence of alcohol consumption habit has highly significant risk for development of OSCC; however the studies shows moderate heterogeneity (*I*^2^ = 40–50%). Certain genes such as HIF (*Z* = 2.82, *p* = 0.05), MTNR1 (*Z* = 12.12, *p* ≤ 0.0001) and DEC (*Z* = 10.46, *p* ≤ 0.0001) shows statistically significant correlation in presence of smoking and chewing habit with very low heterogeneity (*I*^2^ = 0%). The CYP1 gene shows a highly significant (*p* ≤ 0.0001) correlation (*Z* = 3.11) in presence of mixed habit with 0% heterogeneity.

**Conclusions:**

Asian countries show a large cluster of patients with a genetic risk for development of OSCC. Genetic factors such as P53, CASP 8, HIF, DEC1, MTNR1 and CYP1A1 show statistically significant risk for development of OSCC in the presence of risky environmental factors such as tobacco and alcohol.

## Plain language summary

Various behaviors, such as smoking and drinking alcohol, increase the likelihood of developing oral squamous cell carcinoma, a type of mouth cancer. However, many individuals who smoke or drink alcohol do not develop this cancer. We investigated the role of inherited, genetic factors in people who develop the cancer. We looked at research studies published between January 2000 and April 2024. We found various genetic differences that can increase the likelihood of developing oral squamous cell carcinoma in people who use tobacco or alcohol. This information could be presented to the public so they better understand the impact of using tobacco and alcohol consumption on development of oral cancers. Also the genetic differences should be further studied to determine whether targeted strategies to prevent cancers could be introduced.

## Abstract (web summary)

Jadhav K et al. systematically review and analyse genetic susceptibility for development of oral squamous cell carcinoma. Asian populations show high genetic susceptibility with genetic factors such as P53, CASP 8, HIF, DEC1, MTNR1 and CYP1A1 polymorphism increasing risk in the presence of risky behaviors such as using tobacco and alcohol.

## Introduction

Oral squamous cell carcinoma (OSCC) commonly termed as oral cancer, oral cavity cancer is the most common form of cancer in head and neck region (HNSCC). Development of OSCC is a multistep process involving both genetic as well as many environmental risk factors. Betel quid chewing, tobacco use, and alcohol consumption are the common environmental risk factors^1^. However, despite exposure to the same environmental factors, some individuals only develop OSCC, indicating that genetic factors also play a critical role. The presence of these environmental risk factors and certain gene polymorphisms may increase OSCC susceptibility^1^. The identification of susceptibility-related genes for screening the high-risk individuals for increased predisposition to cancer is very important for the prevention of OSCC.

Single nucleotide polymorphism (SNP), can alter any gene expression. SNP arises due variation in DNA nucleotide sequence (A, T, C or G) changes more than 1% within a population. Previous studies have reported that SNPs located within a promoter or other regulatory regions of genes are associated with the development of certain diseases^2^, and several SNPs have been reported as predictive factors for a high OSCC risk^3^. The genetic abnormalities of human cancer to a certain extent depend on geographical location, cultural and environmental backgrounds^4^.

An individual’s genetically coded capability to cope with the carcinogens probably determines who experiences cancer and who does not^5^. The biological mechanisms like DNA repair, apoptotic pathway, and immune check along with certain biological processes like detoxification, biotransformation, and elimination of procarcinogens are the major factors which determine the occurrence of tobacco–alcohol induced HNSCC^6,7^. It should be stressed that structural polymorphic variants exist in the genes that code for the enzymes catalyzing the reactions in the above-mentioned defense mechanisms. A genetic polymorphism may alter the activity of the enzyme encoded by a polymorphic gene, thereby determining the differences in individuals’ responses to carcinogens and in their susceptibility to cancer^8–10^. Certain form of cancers like breast cancer, colon cancer, ovarian cancer arises due to known single gene abnormality, but in case of head and cancer which is a multifactorial disease the environmental risk factors must be present in addition to risk bearing genotype^11^. In the event of HNSCC, an individual’s genetic susceptibility most likely comes from a combination of several unfavorable but rather common genetic polymorphisms^11,12^. Besides the above-mentioned structural variations in the DNA chain, some modifying effects of epigenetic variations in carcinogenesis have recently been recognized^13^. The genetic and epigenetic variations probably explain most of the difference in inter-individual susceptibility to many types of disease, including cancer^14–16^.

Large family studies conducted by Goldgar et al., Foulkes et al. showed that there is three to eight fold increased risk of  HNSCC in first degree relatives^17,18^. Based on evidence from molecular and epidemiological studies the concept of genetic susceptibility for HNSCC can be proved. Emerging data from case-control studies of several phenotypic and genotyping assays support the hypothesis that genetic susceptibility plays an important role in the etiology of HNSCC. This hypothesis supports the fact that inherited differences in the efficiencies of carcinogen metabolizing systems like DNA repair systems, cell cycle control or apoptosis systems, or a combination of these factors influences one’s risk for tobacco-induced cancers. By use of biomarker assays if we can identify such individuals at risk of developing HNSCC, it will have profound influence on primary prevention, early detection and secondary prevention strategies^19^.

The literature search revealed that there is voluminous literature with multiple set of genes have been studied to correlate the role of genes in susceptibility for development of head and neck cancer. Most of the studies have tried to establish the statistical correlation between SNP and environmental risk factors for development of OSCC. Few studies have shown the importance of epigenetic markers like micro RNA in susceptibility for development of OSCC. Although there are many individual studies focusing on this aspect it is now time to analyze this vast literature in order to understand the influence of any particular gene or group of genes over susceptibility for OSCC. Although there are many studies focusing environmental risk factors and genetic susceptibility for development of OSCC; there is lack of any analytical study focusing on geographical and racial variation along with risk factors in genetic susceptibility of OSCC. Through this review we have systematically reviewed and analyzed variations in genetic susceptibility in terms of geographical areas, ethnic background of people, habit related risk factors.

The present review study has revealed that Asian population showing high genetic susceptibility is at high risk for development of oral cancer. Risk factors like tobacco and alcohol influences the genes responsible for development of oral cancer. Study results shows genetic factors like P53, CASP 8, HIF, DEC1, MTNR1 and CYP1A1 polymorphism has significant risk for development of OSCC in presence of risky factors like tobacco and alcohol.

## Methodology

This review protocol has been registered under PROSPERO (International prospective register for systematic review by NIHR) CRD42023422519. Institutional review board (IRB) approval was not obtained for this review as the analysis is based on already published articles.

### Review question

The review question was formulated based on the PICO format. The question was “does any particular gene or group of genes have a higher influence over the susceptibility of  OSCC”? In this review question, the population was all patients diagnosed with  OSCC. The intervention was genes and/or a group of genes related with susceptibility of OSCC. Since this is an observational review, the odds ratio of association was considered as the standard measure for the genes expression. The role of genes or genetic factors was compared with the role of environmental risk factors like tobacco and alcohol for the development of OSCC. The outcome was susceptibility for development of OSCC. As some studies have used the term ‘risk of development of OSCC’, it was also included in the outcome of this review.

### Search strategy

English language literature published from 2000 to April 2024 was searched for relevant data through various databases. These include electronic databases Medline (through OVID), Web of Science, Science Direct, EMBASE, and Cochrane, Scopus. The articles were searched by using appropriate medical subject headings (MeSH Terms). The keywords with bullion used for search of the articles are Head and neck cancer, Oral cancer, Oral squamous cell carcinoma, genetic susceptibility, genetic association, epigenetic factors. The observational studies (case control, cross-sectional, and cohort) involving lab experiments to assess the association between gene expression and susceptibility for OSCC were considered for this review.

The original, experimental, laboratory studies were considered for this review. The review studies, letter to the editor, conference proceedings, and short communications were not considered in the review. Wherever the full text was not available, the corresponding authors were contacted for full texts. The unpublished data that include ahead of print publications, e-publications are included in this review. The gray literature, dissertations, thesis, etc., were not considered for review.

### Inclusion criteria

1. Studies involving patients histologically diagnosed with OSCC.
2. Studies involving patients with tobacco-associated OSCC.
3. The studies showing a clear association/correlation between a particular gene and group of genes with susceptibility or risk of development of OSCC.
4. The studies calculating the odds ratio or the fold change as a statistical measurement for association.

### Exclusion criteria

1. Studies involving Oral cancer other than OSCC.
2. Studies with recurrence of OSCC or HPV associated OSCC.
3. Studies with insufficient data regarding gene expression analysis.
4. Studies assessing the prognosis or outcome.

### Quality appraisal

The studies were selected with strict inclusion and exclusion criteria. Studies involving patients histologically diagnosed with oral cancer were only considered for data extraction. Studies with real-time PCR experiments carried out for assessment of gene expression were given priority for inclusion in data synthesis. Studies with odds ratio of association of gene/genes expression specifically for oral cancer/OSCC only were included for data synthesis.

### Data searching and screening

One of our team member (SKN) used the Ovid platform to search all databases using Mesh Terms. The searched results were exported to Rayyan software for the data filtration as per inclusion and exclusion criteria. The remaining four reviewers (KJ, DP, SG, and ShG) screened all the articles that were searched through the Ovid platform. Initially, 1969 articles were exported to Rayyan software. All four reviewers independently screened the title and abstract of all 1969 articles, and based on inclusion and exclusion criteria, 182 articles were selected for the data extraction. Seven articles were added for the extension period from September 2023 to April 2024.

### Data extraction

The data extraction sheet (Supplementary Form 1) was prepared after a discussion among all the team members. It was structured as per the objectives of the present systematic review. All 189 articles were divided among four reviewers (KJ, DP, SG and ShG) for the independent data extraction.

The qualitative data from the selected studies include the following

- The demographic data: patient’s age, gender, ethnicity, geographical location.
- The clinicopathological data: lesion site, environmental risk factors like habit of tobacco and alcohol consumption, histopathological diagnosis and histological grade of the lesion, and sample type used for assessment of gene expression.
- Studies involving HNSCC, the data regarding OSCC or Oral cavity cancer only was considered in this review.
- Data pertaining HPV associated OSCC or Oral cavity cancer was not considered in this review.
- The molecular data: the genes involved, the method of gene expression analysis (QPCR), polymorphism of single gene or group of genes, allele modification,

The quantitative data include:

The odds ratio for the association of gene polymorphism and the risk of development of OSCC at 95% confidence interval.

The risk of bias assessment was carried out for each included study by using the Newcastle Ottawa scale (Supplementary Form 2). The domain and subdomain parameters were modified as per the review protocol. After data synthesis, out of 189 articles, 16 articles were excluded from further statistical analysis because they lacked sufficient data. So, data from 173 articles was subjected to metanalysis. The studies with a score of eight and above were considered high-quality and considered for further analysis.

### Statistical analysis

The data was initially organized using Microsoft Excel, separating information for genes and calculating odds ratios (OR) to assess susceptibility associations (Supplementary Data 1). Separate data sheets were then prepared for each gene, comparing its expression in cases and controls from the studies included in the analysis (Supplementary Data 2). To study the influence of environmental risk factors (tobacco smoking, tobacco chewing, alcohol and mixed habit) and gene alterations for susceptibility a separate subgroup data sheet was prepared (Supplementary Data 3). Due to the heterogeneity observed across studies for different genotypes and alleles, a random-effects model analysis was applied. The odds ratio (OR) was used as the primary effect measure. All statistical analyses were performed using Review Manager (RevMan) version 5.4. A 95% confidence interval was used for all analyses, and a p-value of ≤0.05 was considered statistically significant. None of the review team members (JK, SoG, DP, ShG, and KSN) were involved in the statistical analysis. It was conducted independently by a statistician (SN), who was not part of the data synthesis process.

## Results

### Overview of studies included in meta analysis

After a careful search with appropriate keywords using the Ovid platform for the period of January 2000 to April 2024, the total articles searched were 1969. The screening results were exported to the Rayyan platform. All the review team members (KJ, DP, SoG, and ShG) had access to article titles through the Rayyan platform. Through blinding mode on, all the members could screen all the titles independently. All the article titles were segregated into three categories: ‘included’, ‘maybe included’ and ‘excluded’. All the members then interacted with each other through online communication mode by switching off the blinding mode in Rayyan platform. During the interaction all the articles were segregated into either inclusion or exclusion category. The articles were included after consensus among all the members of the review team. The differences among opinions were resolved after discussion with advisor member (SKN).

**Fig. 1:**
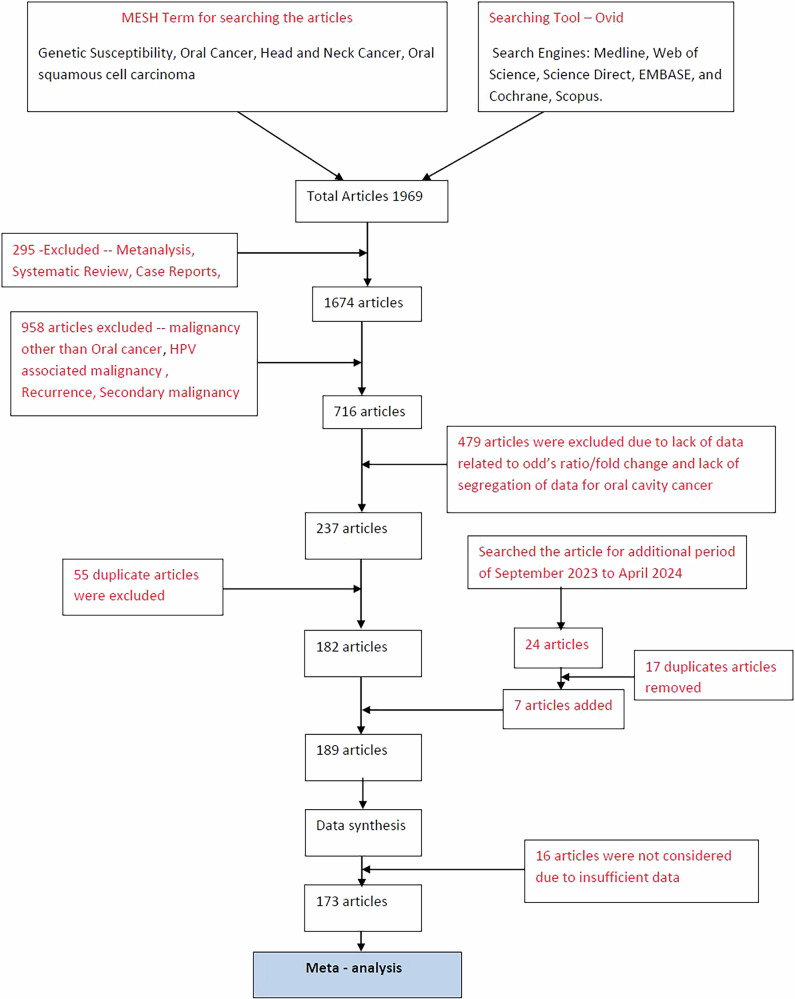
Prisma flow chart.

The flow of exclusion of articles is depicted in the Prisma flow chart (Fig. 1). After exclusion of 295 articles with meta analysis, systematic review and case reports, the 1674 articles were remained. On further screening, 958 articles were excluded because of reasons such as malignancy reported other than oral cavity, HPV associated malignancy, recurrence, and secondary malignancy. The remaining 716 articles were further screened for the presence of measurable outcome, that is, odds ratio (OR). This process resulted in the exclusion of 479 articles. The remaining articles were further assessed for duplicate titles using Rayyan software. The 55 duplicate titles were excluded, so 182 articles remained for further analysis. But since we had exceeded 6 months from the last period of search (September 2023), we decided to search and add the articles for additional period from September 2023 to April 2024. For this additional period, 24 article titles matched our keywords, out of which 17 duplicate titles were removed and the remaining 7 articles were added to the previous search. A total of 189 articles were considered for data synthesis.

### Data extraction

The full text of the 189 selected articles was obtained and uploaded onto the Rayyan platform. The data extraction proforma was prepared and customized as per the outcome expected (Supplementary Form 1). Each article’s full text was opened and discussed through online communication among four members (KJ, DP, SoG, ShG) of the reviewer team for extraction of the required data. For the articles which were showing head and neck cancer (HNSCC), the data pertaining to the oral cavity only was included during data extraction. The quality of articles and risk of bias were assessed using the Newcastle Ottawa scale for case control and cohort studies^20^. The scale was customized keeping the standard domains (Supplementary Form 2). Each article was assessed through the categories of selection, comparability and outcome. The good-quality studies were further included in data sheets. (Supplementary Data 4).

## Overview of results

### Geographical influence over genetic susceptibility

The data sheet (Supplementary Data 1) showed the most number of published studies in area of genetic susceptibility for development of OSCC are from Taiwan^21–65^, followed by India^66–95^, China^96–113^, and USA^114–142^. The studies from other countries are also contributing in literature significantly are Argentina^143,144^, Belgium^145^, Brazil^146–154^, Canada^155^, France^156^, Germany^157^, Greece^158–160^, Indonesia^161^, Iran^162,163^, Italy^164^, Japan^165,166^, Korea^167^, Netherland^8^, Pakistan^168,169^, Poland^170–172^, Republic of Korea^173^, Serbia^174–176^, Spain^177,178^, Thailand^179^, Turkey^180–182^, United Kingdom^183^. Most studies from Taiwan showed an average of 0.5–1.00 odds ratio for the genetic risk of the development of OSCC, whereas from China and India, most of the studies are showing an average of 0.5–2.00 odds ratio for the development of OSCC.

**Fig. 2:**
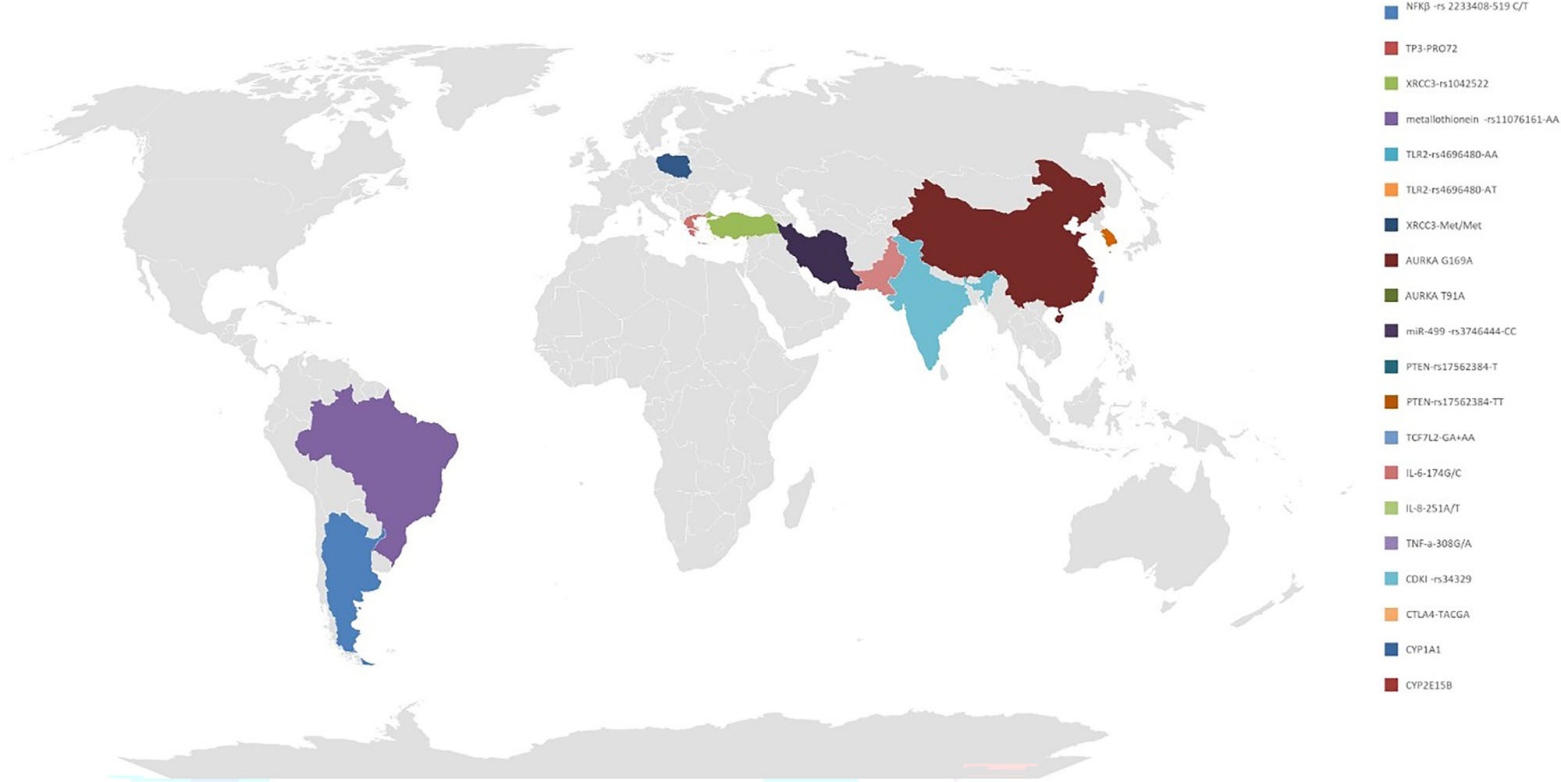
World map graph showing geographical distribution of genes (with odds ratio more than 3.0) associated with susceptibility for development of oral squamous cell carcinoma. *All the genes cannot be shown in the graph, so please refer Table 1.

Many studies from India revealed higher genetic risk of more than 3.00 odds ratio for the development of OSCC. Among these studies, the genes involved are SND1 (OR = 34.1)^67^, TCF7L2 (OR = 12.16)^67^, CYP1A1 (OR = 12.0)^70^, GSTM1 (OR = 4.25)^70^, GSTM1 (OR = 3.2)^72^, IGF2 (OR = 4.6)^73^, CTLA4 (OR = 7.72)^77^, miRNA 149 (OR = 14.1)^80^, CYP1A1 (OR = 5.28)^90^ and GSTT1 (OR = 6.49)^92^. Among these studies only two studies (Kaur et al.^73^, Shreelekha et al.^90^) has sample size less than hundred patients. Few studies from China showed an odds ratio of more than 3.00, and the genes involved are AURKA (OR = 4.77)^97^, PTEN (OR = 3.68)^103^, IL 4 promoter (OR = 6.00)^109^, miR-499 (OR = 3.154)^112^. Among these studies one study (Huang et al.^97^) has sample size less than hundred patients. Taiwan studies showing an odds ratio of more than 3.0 are involving CYP1A1-A/G (OR = 5.08)^24^, CYP1A1-G/G (OR = 18.16)^24^, HIF1ɑ (OR = 3.36)^27^ and miRNA-499 (OR = 4.52)^37^, MMP 14 (OR = 7.08)^40^, Ecadherin (OR = 3.64)^57^. All these studies has sample size more than hundred patients. Some studies from other countries like Argentina (TP53, XRCC3), Brazil (metallothionein, XRCC3, TLR2), Greece (IL 6,8 and TNFɑ), Iran (lncRNAH19, MTHFR), Korea (CYP1A1), Pakistan (GSTM1, GSTT1 and CYP1A1), Poland (TC2, E2F2, LTF), Republic of Korea (ADH1B), Turkey (EGF) are also showing an odds ratio of more than 3.0 for association between abnormal genes and risk of OSCC (Fig. 2 and Table 1). Interestingly, none of the studies from the USA showed a high risk (OR ≥ 3.0) of genetic susceptibility for the development of OSCC.

**Table 1:** Geographical distribution of genes (with odds ratio more than 3.0) associated with susceptibility for development of oral squamous cell carcinoma

Country	Gene	Odds ratio	Country	Gene	Odds ratio
Argentina	TP3-PRO72	9.8	Iran	LncRNA H19 rs217727 -TT Dominant	6.04
	XRCC3-rs1042522	3.14		LncRNA H19 rs217727 -TT-Recessive	5.32
	NFKβ -rs2233408-519 C/T	3.1		Mthfr-C677T-TT	3.1
Brazil	metallothionein -rs11076161-AA	4.53	Korea	CYP1A1-m2/m2	3.3
	XRCC3-Met/Met	3.3	Pakistan	GSTM1/GSTT	3.66
	TLR2-rs4696480-AT	5.31		GSTM1 and GSTT1 both null	9.26
	TLR2-rs4696480-AA	6.64		GSTM/GSTT null and CYP1A1 wild type	4.5
China	AURKA T91A	3.1		GSTM/GSTT either null and CYP1A	5.593
	AURKA G169A	4.77		GSTM1 + GSTT1 + CYP1A1	16.1
	miR-499 -rs3746444-CC	3.154	Poland	TC2 C776G-G/G	4.38
	PTEN-rs17562384-T	3.68		E2F2-rs6667575-A/G	127.51
	PTEN-rs17562384-TT	3.5		LTF-rs4637321-AG	4.41
	IL4 Promoter -CC	6		LTF-rs4637321-GG	6.25
Greece	IL-6-174G/C	8.33		LTF-rs4637321-AG + GG	5.45
	IL-8-251A/T	3.54	Republic of Korea	ADH1B	3.55
	TNF-a-308G/A	15.27	Taiwan	HIF-1 ɑ G1790A-AA	3.36
India	CYP2E15B	4.26		HIF-1 ɑ G1790A-AA	3.1
	CYP1A1	5.28		MMP14	7.08
	SND1-r s7778413-CC	34.1		E cadherin −347-G/GA	3.64
	TCF7L2-rs7085532	3.45		E cadherin −347-GA/GA	3.77
	TCF7L2-AA	12.16		SULT1A1	3.24
	TCF7L2-GA	4.73		miRNA499- rs3746444-CC	4.52
	TCF7L2-GA + AA	6.85		CYP1A1-A/G	5.08
	CYP1A1	12		CYP1A1-G/G	18.16
	GSTM1	4.25	Turkey	EGF + 61 A > G-GG	6.93
	CDKI -rs34329	3.46			
	Retinoblastoma 1-1-rs3092904	3.46			
	GSTM1	3.2			
	Poly (ADP-Ribose) -rs1136410-CC	3.98			
	Poly (ADP-Ribose) -rs3219090-CC	3.19			
	GST1-Null	6.25			
	GSTM1 and GSTT1 Null	5.27			
	CTLA4-TACGA	7.72			
	TLR9-rs5743836-CC	3.939			
	miR-196a2 -rs11614913-CC	4.51			
	miR-149- rs2292832-TT	14.1			

### Racial/ethnicity influence over genetic susceptibility

The data sheet (Supplementary Data 1) for racial/ethnicity and genetic abnormality for the development of OSCC showed the Han population is at high risk of the development of OSCC. A total of 33,515 OSCC patients’ data revealed an average odds ratio of 1.37 for genetic susceptibility. A total of sixty-five genes have been studied in association with genetic susceptibility for oral cancer development among the Han population. Among these, certain genes like AURKA^97^, Cytochrome P450^24^, E-cadherin^47^ and Interleukin 4 promoter^101^ showed more than three odds ratio (OR ≥ 3.0) of association between gene abnormality and the development of oral cancer. The other ethnic populations like Indo Aryan, Non-Hispanic and White and mixed population also showed higher susceptibility as compared to other ethnic groups. Indo Aaryan population showed a total number of 7561 OSCC patients studied with an average odds ratio of 1.6 for genetic susceptibility. The non-Hispanic population showed a total number of 7129 OSCC patients studied with an average of 1.06 odds ratio for genetic susceptibility. The white and mixed population showed a total number of 1123 OSCC patients with an average of 1.3 odds ratio of genetic susceptibility.

### Genetic abnormality leading to susceptibility for the development of OSCC

**Fig. 3:**
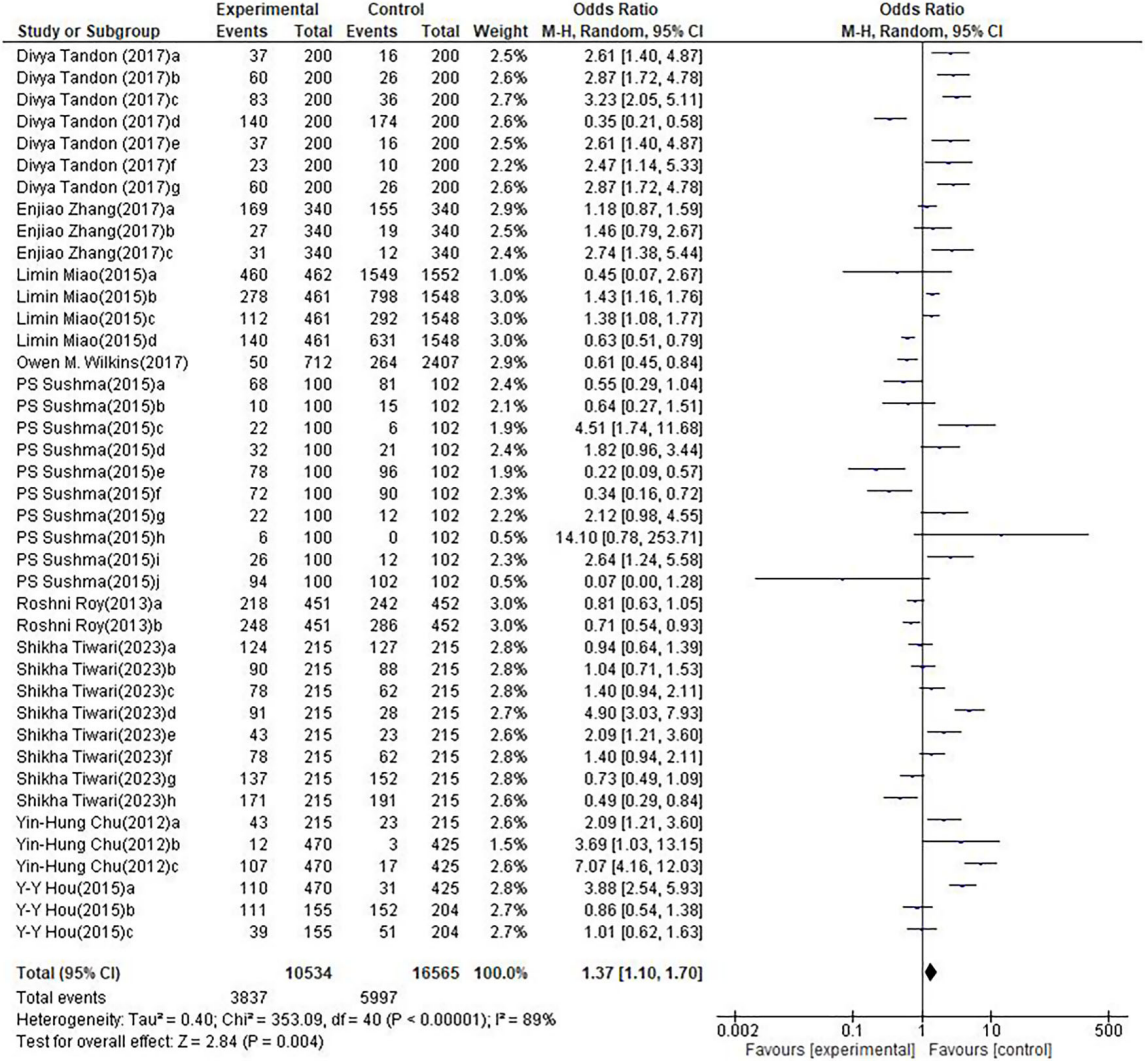
Forest plot for miRNA random effect analysis (*n* = 3837) at 95% CI with overall effect *Z* = 2.84 showing statistically significant correlation (*p* = 0.004).

**Fig. 4:**
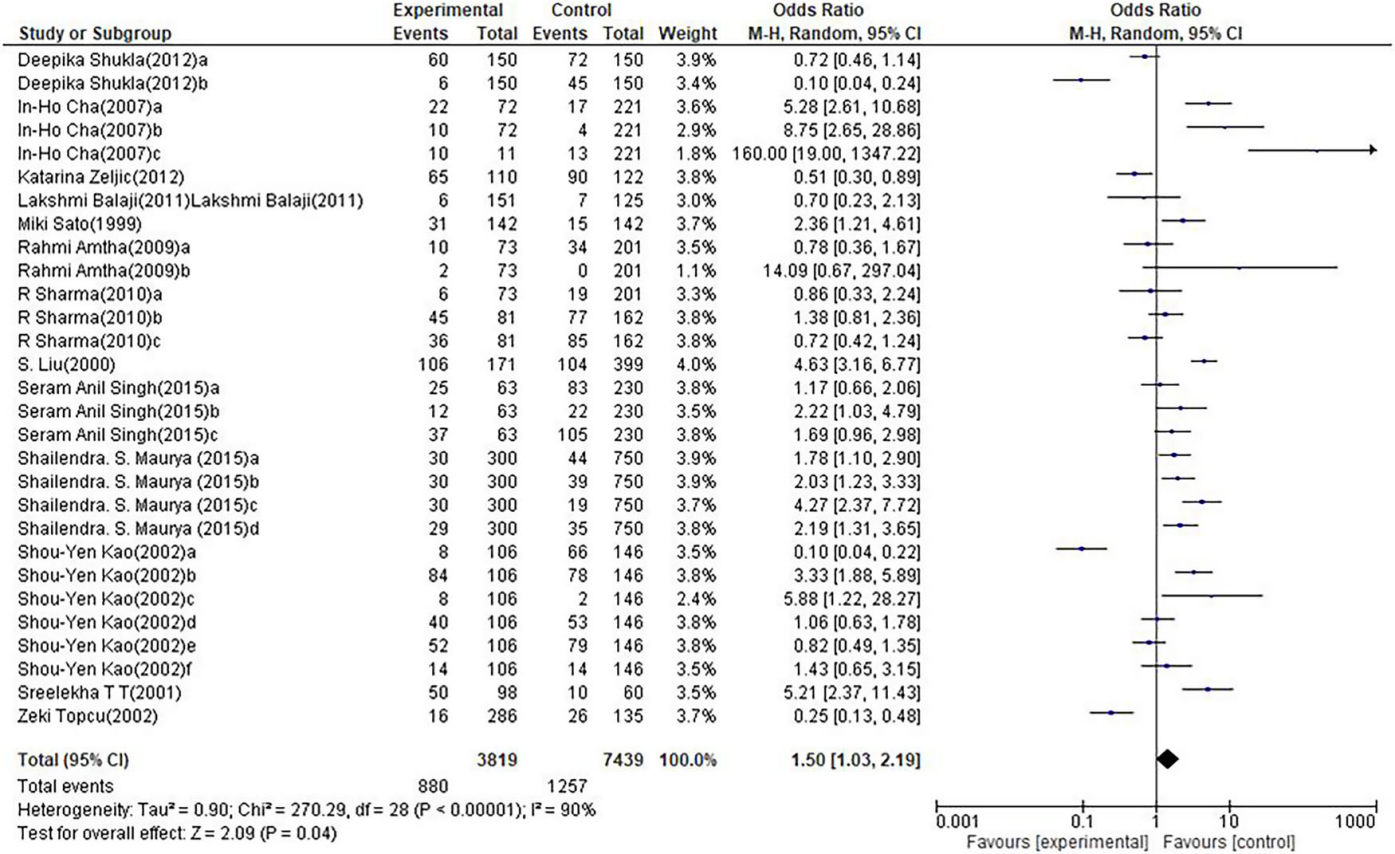
Forest plot for CYP1A1 random effect analysis (*n* = 880) at 95% CI with overall effect *Z* = 2.09 showing statistically significant correlation (*p* = 0.04).

**Fig. 5:**
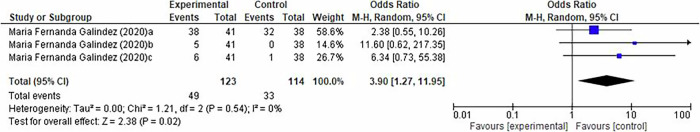
Forest plot for NFKß random effect analysis (*n* = 49) at 95% CI with overall effect *Z* = 2.38 showing statistically significant correlation (*p* = 0.02).

The random effect model analysis was carried out to assess the association between abnormal genes and genetic susceptibility for the development of OSCC (Supplementary Figs. 1–28). The heterogeneity test was also carried out to check for heterogeneity (*I*^2^) among included studies for particular abnormal genes. It was observed that miRNA (*Z* = 2.84) (Fig. 3), CYP1 (*Z* = 2.09) (Fig. 4), showed statistically significant (*p* < 0.05) association with genetic susceptibility and risk for development of OSCC. However, these results showed significant heterogeneity among the studies (*I*^2^ ≥ 90%). The substantial heterogeneity warrants careful consideration of these results. Some of the other genes {(UGT (*Z* = 1.55), MGMT (*Z* = 0.86), WISP (*Z* = 0.13), and Survivin (*Z* = 0.39)} showed no heterogeneity (0%) among the studies but did not show statistically significant (*p* ≥ 0.05) association. The NFKß gene showed no heterogeneity (0%) and statistically significant (*p* = 0.02) association (*Z* = 2.38) with genetic susceptibility for the development of OSCC (Fig. 5 and Table 2).

**Table 2:** Summary of random effect model analysis showing affected genes associated with susceptibility for development of oral squamous cell carcinoma

Sr. no.	Gene	*Z* (Association score)	Heterogeneity (*I*^2^)	Significance (*p*)
1	TNF	1.48	93%	0.14
2	P73	0.89	99%	0.37
3	miRNA	2.84	89%	0.004
4	CYP1	2.09	90%	0.04
5	MTHFR	0.15	92%	0.88
6	GSTM	0.59	91%	0.56
7	UGT	1.55	0%	0.12
8	Interleukin	0.71	90%	0.48
9	VEGF	1.63	78%	0.10
10	MMP	0.67	74%	0.50
11	Ecadherin	1.66	90%	0.10
12	CASP8	0.70	94%	0.48
13	XRCC1	0.96	73%	0.34
14	MGMT	0.86	0%	0.39
15	P53	0.06	97%	0.95
16	ADAM1	1.39	93%	0.17
17	LTF	0.51	74%	0.61
18	MT	1.01	54%	0.31
19	XPD	0.97	70%	0.33
20	NFKbeta	2.38	0%	0.02
21	IGF2	0.61	99%	0.54
22	lncRNA	1.37	78%	0.17
23	TLR	0.45	14%	0.66
24	WISP	0.13	0%	0.89
25	COX2	0.56	98%	0.57
26	Survivin	0.39	0%	0.70
27	RAD5	0.54	92%	0.59
28	ADH1C	1.05	97%	0.29

*p* ≤ 0.05 is considered statistically significant.

### Subgroup analysis for assessment of effects risky habits and genetic susceptibility

**Fig. 6:**
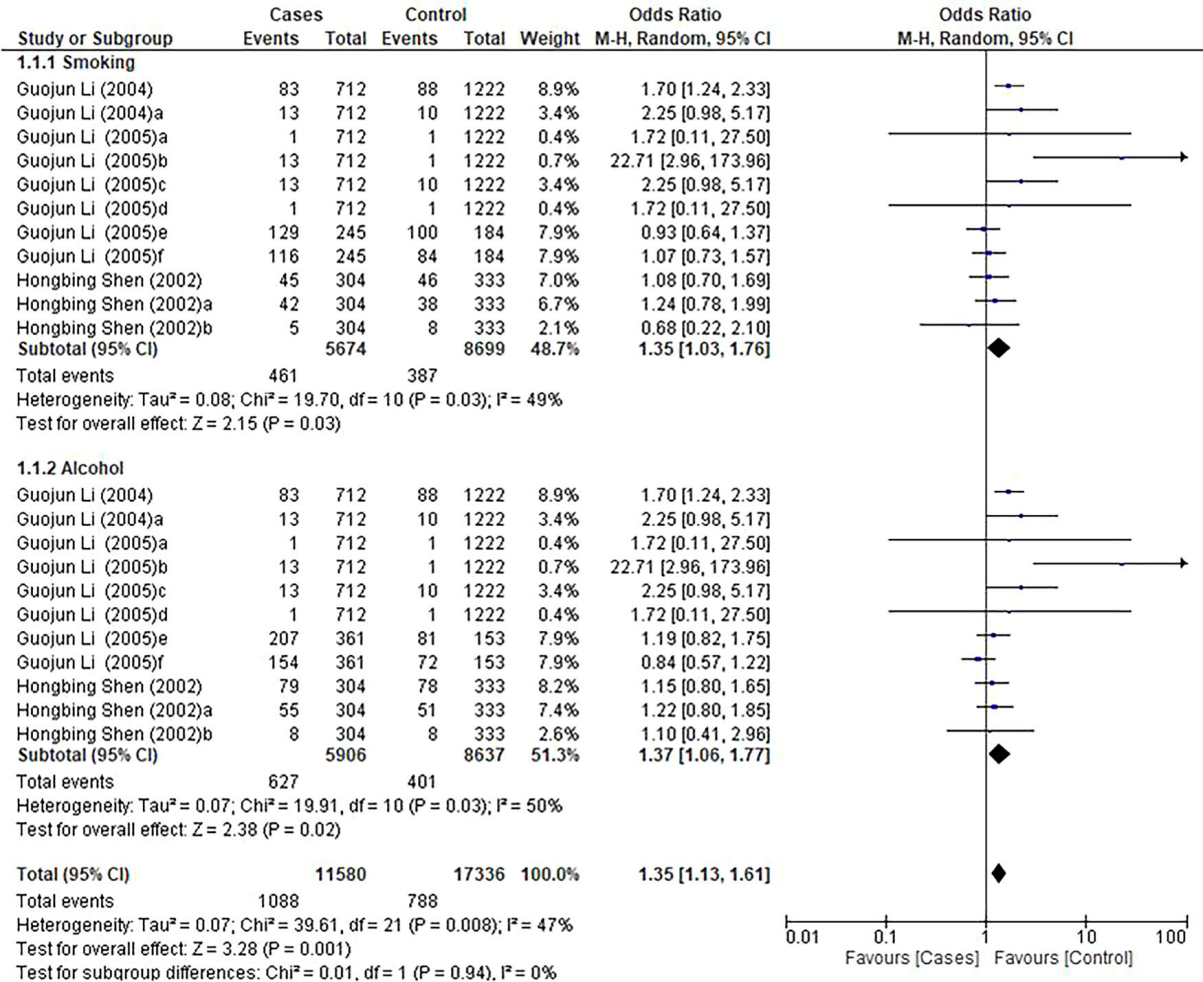
Forest plot for p^53^ random effect analysis for smoking (*n* = 461) at 95% CI with overall effect *Z* = 2.15 showing statistically significant correlation (*p* = 0.03). Random effect analysis for alcohol (*n* = 627) at 95% CI with overall effect *Z* = 2.38 showing statistically significant correlation (*p* = 0.02).

**Fig. 7:**
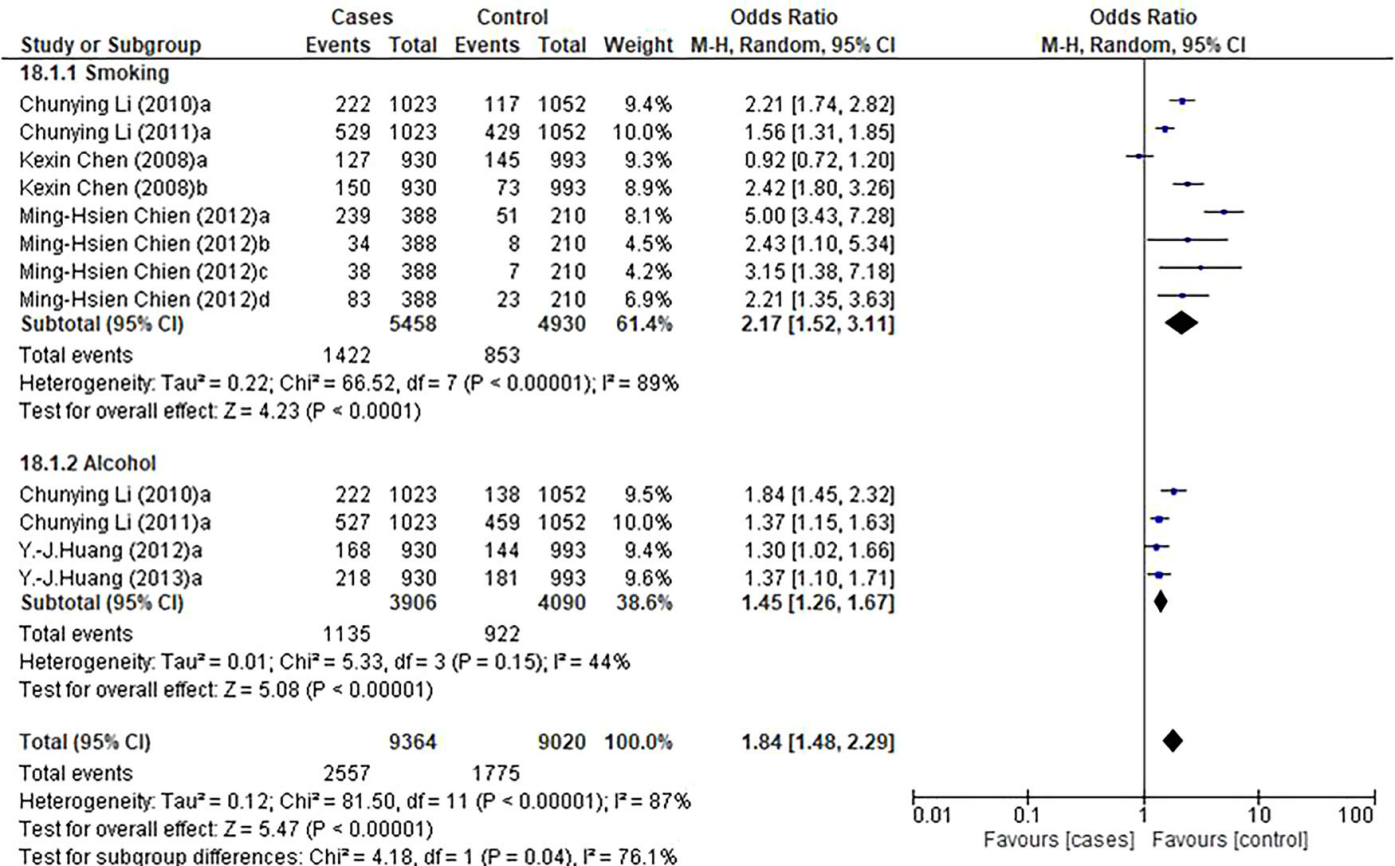
Forest plot for CASP8 random effect analysis for smoking (*n* = 1422) at 95% CI with overall effect *Z* = 4.23 showing statistically significant correlation (*p* ≤ 0.0001). Random effect analysis for alcohol (*n* = 1135) at 95% CI with overall effect *Z* = 5.08 showing statistically significant correlation (*p* ≤ 0.0001).

**Fig. 8:**
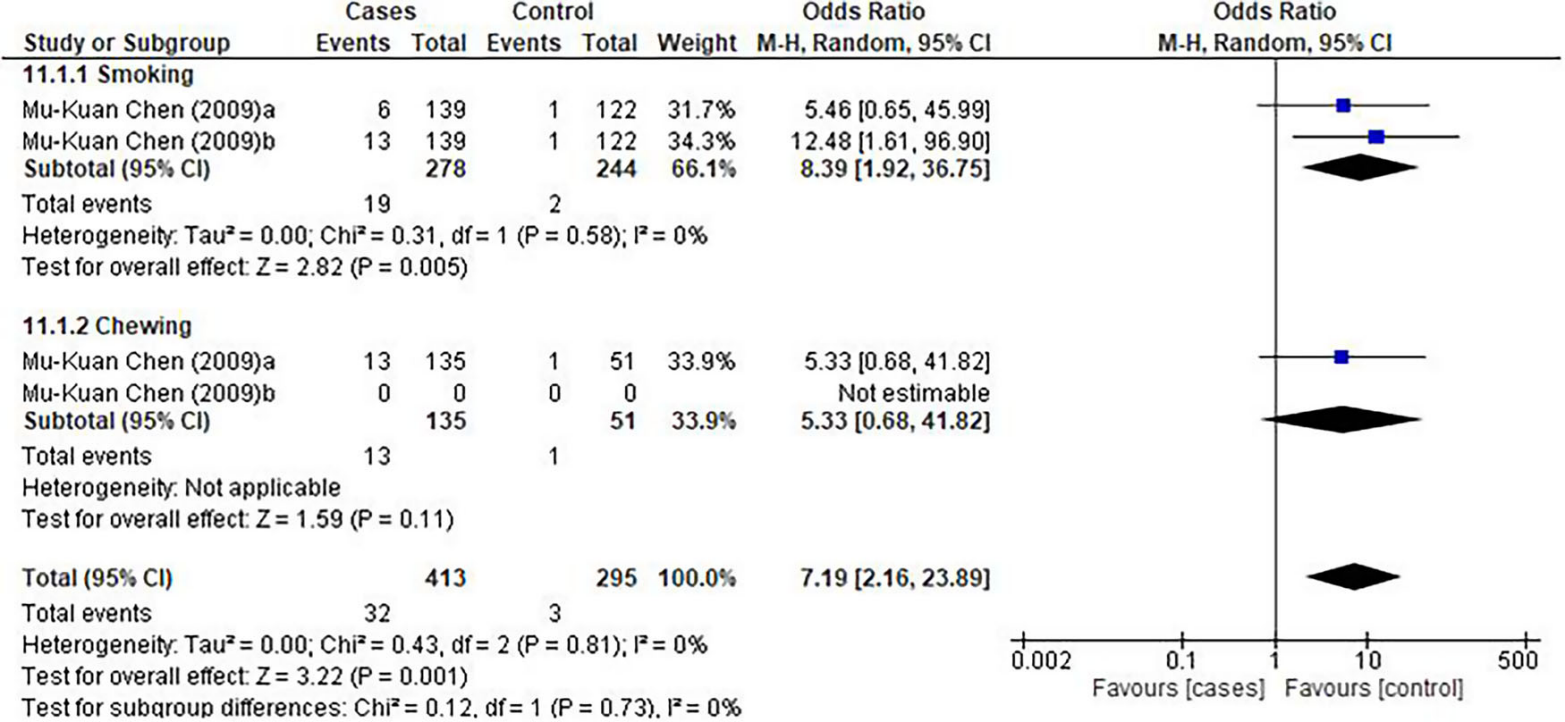
Forest plot for HIF random effect analysis for smoking (*n* = 19) at 95% CI with overall effect *Z* = 2.82 showing statistically significant correlation (*p* = 0.005). Random effect analysis for chewing tobacco (*n* = 13) at 95% CI with overall effect *Z* = 1.08 showing statistically not significant correlation (*p* = 0.11).

**Fig. 9:**
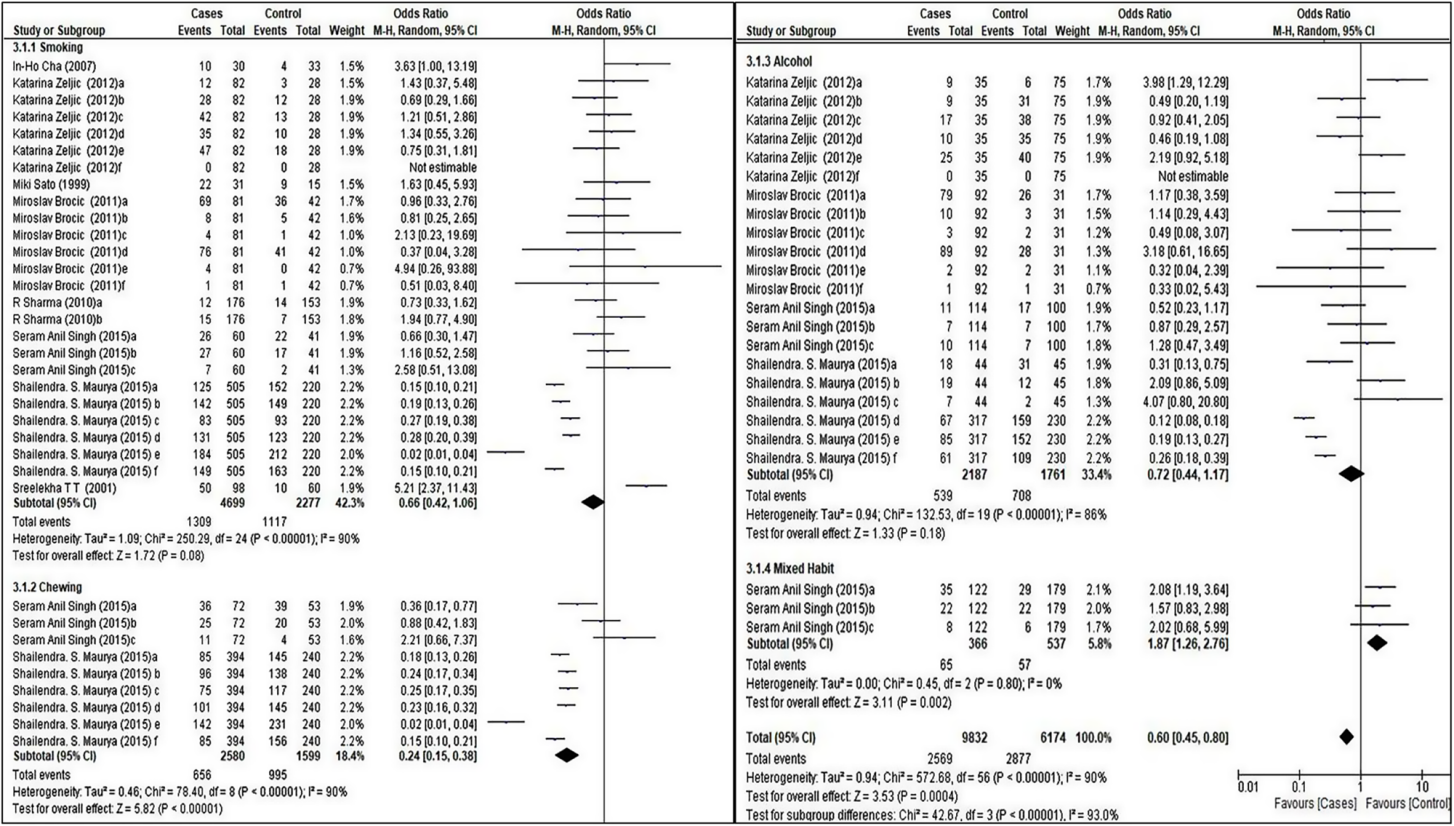
Forest plot for CYP1A1 random effect subgroup analysis for smoking (*n* = 1309) at 95% CI with overall effect *Z* = 1.72 showing statistically not significant correlation (*p* = 0.08). For chewing tobacco (*n* = 656) at 95% CI with overall effect *Z* = 5.82 showing statistically significant correlation (*p* ≤ 0.0001). For alcohol (*n* = 539) at 95% CI with overall effect *Z* = 1.33 showing statistically not significant correlation (*p* = 0.18). For mixed habit (tobacco and alcohol) (*n* = 65) at 95% CI with overall effect *Z* = 3.11 showing statistically not significant correlation (*p* = 0.002).

The meta-analysis was carried out to assess the effect of environmental risk factors like habit of tobacco smoking, tobacco chewing, alcohol and mixed habits (tobacco and alcohol) upon the genetic susceptibility for the development of oral cancer. The analysis showed polymorphism in P^53^ gene in presence of smoking (*Z* = 2.15, *p* = 0.02), alcohol (*Z* = 2.38, *p* = 0.02) and mixed habit (*Z* = 3.28, *p* ≤ 0.001) has highly significant risk for development of oral cancer (Fig. 6). Similarly CASP 8 (*Z* = 5.38, *p* ≤ 0.0001) showed highly significant risk for development of oral cancer in presence of alcohol consumption habit (Fig. 7). Although there is significant results observed with these genetic factors (P^53^, CASP8), but the studies shows moderate heterogeneity (*I*^2^ = 40–50%). Some genes HIF (*Z* = 2.82, *p* = 0.05) (Fig. 8), MTNR1 (*Z* = 12.12, *p* ≤ 0.0001) and DEC (*Z* = 10.46, *p* ≤ 0.0001) showed statistically significant correlation in the presence of smoking and chewing habit with very low heterogeneity (*I*^2^ = 0%) among the studies. These results are having very low heterogeneity may be because of single study involved in analysis. Interestingly, the CYP1A1 gene showed a highly significant (*p* ≤ 0.0001) correlation (*Z* = 3.11) (Fig. 9) in the presence of mixed habits (tobacco and alcohol) with genetic susceptibility for the development of Oral cavity cancer (Table 3).

**Table 3:** Subgroup analysis showing effect of risky environmental factors over gene alteration leading to susceptibility for development of oral squamous cell carcinoma

		Smoking			Chewing			Alcohol			Mixed habit		
Sr. no.	Gene	*Z*	(*I*^2^)	*p* value	*Z*	(*I*^2^)	*p* value	*Z*	(*I*^2^)	*p* value	*Z*	(*I*^2^)	*p* value
1	TNF	8.30	78%	<0.00001	0.90	0%	0.37	1.32	83%	0.19			
2	P73												
3	miRNA	0.95	94%	0.34	1.05	96%	0.29	1.10	95%	0.27			
4	CYP1	1.72	90%	0.08	5.82	90%	≤0.0001	1.33	86%	0.18	3.11	0%	0.002
5	MTHFR												
6	GSTM	24.32	94%	<0.00001	25.94	96%	<0.00001	22.73	94%	<0.00001			
7	UGT	3.80	91%	0.0001				2.30	47%	0.02			
8	Interleukin	0.88	87%	0.38	1.20	67%	0.20	1.65	65%	0.10			
9	VEGF												
10	MMP	0.10	84%	0.92	0.12	64%	0.90	0.26	49%	0.80			
11	Ecadherin	1.95	91%	0.05	0.53	94%	0.60						
12	CASP	4.23	89%	<0.00001				5.08	44%	<0.00001			
13	XRCC1	0.99	98%	0.32	1.23	98%	0.22	11.70	NA	<0.00001			
14	MGMT												
15	P53	2.15	49%	0.03				2.38	50%	0.02	3.28	47%	0.001
16	ADAM1	0.00	0%	1.00	0.00	0%	1.00						
17	lncRNA	1.13	93%	0.26				0.02	20%	0.98			
18	TLR												
19	WISP												
20	COX2	1.66	97%	0.10				1.22	98%	0.22			
21	ADH1C	0.02	0%	0.98				1.98	69%	0.05			
22	HIF	2.82	0%	0.005	1.59	NA	0.11						
23	FUT	5.51	89%	<0.00001	0.26	50%	0.79						
24	MTNR1	12.12	0%	<0.00001	4.30	0%	<0.00001						
25	MASPIN	1.45	1%	0.15	0.94	11%	0.34	1.95	73%	0.05			
26	RECK	6.67	65%	<0.00001	0.95	45%	0.05						
27	Leptin	1.05	81%	0.30	1.41	0%	0.16	1.05	82%	0.29			
28	CA9	4.84	61%	<0.00001	1.53	0%	0.13						
29	ALDH1	0.69	87%	0.49				3.64	59%	0.0003			
30	DEC1	10.46	7%	<0.00001				4.42	0%	<0.00001			

*p* ≤ 0.05 is considered as statistically significant.

### Subgroup analysis for assessment of family history and oral mucosal trauma with genetic susceptibility

The analysis shows that the pooled odds ratio for the association between family history and the genetic polymorphism was 0.70 (95% CI: 0.23–2.18, *p* = 0.54), indicating that there was no statistically significant association between family history and the presence of the gene mutation. The confidence interval includes 1, further confirming the absence of a meaningful relationship. The heterogeneity among the included studies was very high (*I*² = 96%, *p* < 0.00001), suggesting substantial variability in the findings across studies. Therefore, the overall evidence does not support a consistent association between family history and polymorphism of genes (Supplementary Fig. 29).

The pooled odds ratio for the association between chronic mucosal trauma and polymorphism of gene was 1.38 (95% CI: 0.28–6.87, *p* = 0.69), indicating that there was no statistically significant association between the two variables. The confidence interval is wide and includes 1, suggesting that the true effect could range from a possible protective to a risk association, but without statistical significance. The heterogeneity among the included studies was very high (*I*² = 95%, *p* < 0.00001), reflecting considerable variability between study results. Hence, based on the combined evidence, there is no consistent or significant relationship between chronic mucosal trauma and genetic susceptibility (Supplementary Fig. 30).

## Discussion

Among the many factors that influence the development of oral cavity cancer, the polymorphism of genes plays a pivotal role. It has been shown that alterations in genes that compromise the ability of the body to remove the constituents of risk factors increase the risk of cancer development. The same applies to the polymorphisms that prevent DNA repair, which is caused by environmental risk factors like tobacco smoking and chewing^184^. Mutation in the genes following exposure to environmental risk factors is a major source for single nucleotide polymorphism (SNP) linked with cancer development. Many SNPs are located within the regulatory region of genes (Promoter, exon, introns), which influences the abnormal expression of genes^185^.

The processes such as biotransformation, detoxification, elimination of procarcinogens along with DNA repair mechanism and apoptosis are probably the most important factors influencing the development of tobacco-alcohol induced head and neck cancer^6,8^. It is now well understood that structural polymorphic variants exists in genes that code for certain enzymes which code for catalyzing the above processes. A genetic polymorphism may alter the activity of enzymes encoded by polymorphic genes, thereby determining the differences in an individual’s response to carcinogens and their susceptibility to cancer^9,10,186^.

In the present study, several genes (TNF, miRNA, CYP1, GSTM, Ecadherin, COX2, IGF2, RAD5 and ADH1c) showed statistically significant correlation with genetic risk for the development of OSCC. TNF and miRNA are epigenetic variations that affect genetic susceptibility. Liu et al. showed through a case-control study that the miRNA binding site of the TNF gene is associated with an increased risk of HNSCC. The genotype rs8126 variant CC and CC/CT genotypes are most frequently associated with an increased risk of HNSCC^187^. The present analysis showed the TNF gene with 308 G/A genotype has higher odds of association for the development of OSCC. These results are seen in the Han and Greek populations. Some study groups have shown that miR-499 and miR-196A2 are significantly associated with the risk of head and neck cancer susceptibility. In the present study, we could also see a higher odds ratio of association of miR-196A2 and miR-499 with the genetic risk of OSCC development. People with polymorphism of miRNA 499 at genotype rs3746444 with CC allele have high risk of OSCC development. Similarly, the polymorphism of miR-196A2 at rs11614913 with C/T allele showed a high risk. As per the study literature, polymorphism of miRNA 499 is commonly seen in the Han population, whereas polymorphism of miRNA 196A2 is common in Indo–Aryan population.

Various case-control studies have shown CYP1A1, CYP2D6, and CYP2E1as risk factors for HNSCC ^188–190^. *CYP1A1* gene codes for aryl hydrocarbon hydroxylase, initiates a multienzyme pathway that activates polycyclic aromatic hydrocarbons, including benzo(a)pyrene, to highly electrophilic metabolites such as Benzo(a)pyrene diol epoxide(BPDE)^188^. *CYP2D6* metabolizes a wide range of nitrogen-containing drugs, including narcoleptics, antidepressants, and ß-blockers, as well as the tobacco-specific nitrosamine 4-(methylnitrosamino)-1-(3-pyridyl)-1-butanone, to mutagenic products^189^. *CYP2E1* is involved in the oxidation and adduct formation of several components of cigarette smoke, such as *N*-nitrosamines and benzene, and contributes to the metabolism of ethanol and acetone^189,190^. The present study analysis showed a highly significant (*p* ≤ 0.0001) relation of polymorphism of CYP1A1 gene and risk (*Z* = 3.11) of development of OSCC in presence of mixed habit of tobacco and alcohol (Table 2). The Indo-Aryan and Han population showed a high odds ratio of association for polymorphism of CYP1A1 gene and risk of development of OSCC.

Several studies in literature have shown the association of GSTM1 null genotype with HNSCC. Most of these case-control studies showed borderline influence when abnormality in individual gene was considered, but when the null genotype of different forms of GSTM1 gene was combined, it showed a significant risk for HNSCC development^188–193^. Present metanalysis showed a statistically significant (*p* ≤ 0.00001) correlation between GSTM1 and genetic risk for the development of OSCC in presence of risky habits. However, heterogeneity among the studies was very high (*I*^2^ = 91%) (Table 2). The present study showed that the GSTM1 null genotype polymorphism is commonly associated with OSCC in the population of India and Pakistan with a higher odds ratio (OR ≥ 3.8) of association (Supplementary Data 1).

In the present analysis, we observed that NFKß gene showed a significant correlation with genetic susceptibility for OSCC. The heterogeneity was nil (*I*² = 0%) and the test for overall effect is statistically significant (*Z* = 2.69, *p* = 0.007). Although this appear to be very promising result but the result is based on only one study having small cohort of patients^143^. This genetic factor is being also studied in Han population. Chen et al. have suggested that either rs28362491 Del/Del or rs72696119 GG polymorphisms were associated with increased risk of Oral cancer^107^.

There is far from consensus on the value of racial information in cancer research. It has been argued that data on racial categories are no longer required in etiological research because they too vague and imprecise^194,195^. Other point to the use of such classification schemes for epidemiological and clinical investigations^196^. With the help of population genetics we now know that race/ethnicity categories correspond to and help identify unique germline alleles and allelic combinations. These ancestral genetic groupings can modify both cancer risk and the molecular subtype of tumors^197^. It is possible that there are complex interactions between ancestry specific genes and race associated environmental exposures. In addition to collecting race/ethnicity data, population specific polymorphisms have been identified that can be used to directly estimate individuals ancestry and this approach could replace self reported ancestry^198,199^.

In this review we could not assess true familial heredity pattern leading to genetic susceptibility for OSCC, may be because sufficient data is not available in the literature to draw some significant results. Huang et al. performed whole genome sequencing to identify genes involved as causative factors for OSCC in single individual family. Their study showed VAV2 and IQGAP1 are the primary causative factors for individual family^200^. Negri Eva et al. have studied the relation between family history and head and neck cancer (HNC). The pooled analysis showed family history of HNC in first-degree relatives increased the risk of HNC (OR = 1.7, 95% confidence interval, CI, 1.2–2.3). The risk was higher when the affected relative was a sibling (OR = 2.2, 95% CI 1.6–3.1) rather than a parent (OR = 1.5, 95% CI 1.1–1.8), The OR rose to 7.2 (95% CI 5.5–9.5) among subjects with family history, who were alcohol and tobacco users^201^. The studies focusing typically familial pattern of heredity and thereby assessing the susceptibility for OSCC can be collected and reviewed separately to understand the true risk of susceptibility for OSCC.

In the present review we found that there is no significant (*p* = 0.69) association between chronic mucosal trauma and genetic polymorphism leading to OSCC development. Although this interpretation is based on small cohort of patients (*n* = 98) with chronic mucosal trauma and showing high heterogeneity (*I*^2^ = 95%). The systematic review and metanalysis by Gupta et al. showed significant association (*p* ≤ 0.00001) between chronic mucosal trauma and OSCC with overall risk ratio of 2.56 at confidence interval of 1.96–3.35. They have stated that chronic mucosal trauma can act as a dependent risk factor in presence of primary risk factors like tobacco and alcohol. The mucosal trauma enhances the carcinogenic effect of tobacco and alcohol^202^. The large cohort study with identification of genetic alteration in OSCC patients with chronic mucosal trauma in presence of risky habit related factors may throw some light on understanding strength of such association.

The geographical and racial/ethnical variations in genetic factors as factors of susceptibility for OSCC can be studied further for confirmation of cause and effect relationship. Linking the high risk genes for particular geographical location, may lead to new pathways and understanding for geographical variation influencing the prevalence of OSCC.

We have followed some deviation from protocol submitted to Prospero. The duration of search was extended from October 2023 to April 2024 so that we could add some more articles in data synthesis. We have added another expert (SN) in the team as he has served as statistician for all analysis involved in this study. Also we could avoid bias in statistical analysis as he was not involved in data synthesis process. In this review we could not assess additional outcome of correlation between positive family history of lesion and genetic susceptibility as the sufficient data is not available in the literature. We have used Newcastle Ottawa scale for risk bias assessment because it is easy to apply and equally effective as compared to Robins e tool.

## Supplementary information

Transparent Peer Review file

Supplementary Information

Description of Additional Supplementary files

Supplementary Data 1

Supplementary Data 2

Supplementary Data 3

Supplementary Data 4

## Data Availability

All the relevant data are available from corresponding author K.J. upon request through mail. Corresponding author will be responsible for replying to all such request. The data include the review protocol submitted for PROSPERO registration in Pdf format and the data extraction files stored in: https://new.rayyan.ai/reviews.

## Code availability

For this systematic review, Raayan software has been used for screening and inclusion and exclusion of articles. All statistical analyses were performed using Review Manager (RevMan) version 5.4.
