# Supplementary material for: Assessment of genetic susceptibility in patients with oral squamous cell carcinoma: a systematic review and meta-analysis
Source: Commun Med (Lond). 2026 Mar 20;6:406. doi: 10.1038/s43856-026-01398-9 (PMC13396505; doi:10.1038/s43856-026-01398-9)

Supplementary Information:

Supplementary Form 1: Data extraction form

Supplementary Form 2: Risk of bias assessment form based on Newcastle Ottawa Scale

Supplementary Figure 1 to Supplementary Figure 28: Results of random effect model analysis showing strength of association between various genetic factors and risk of development of oral squamous cell carcinoma.

Supplementary Figure 29: Results of random effect model analysis showing strength of association between positive family history and risk of development of oral squamous cell carcinoma.

Supplementary Figure 30: Results of random effect model analysis showing strength of association between positive family history and risk of development of oral squamous cell carcinoma.

Supplementary Data Extraction Form 1

Title:

Comments by review authors: *(Please fill this section after the completion of data extraction)*

**Study ID:**		Date form completed:
First author:	Year of study:	Data extractor:
Reference:		Language:
Citation:		

Corresponding Author details:

1. General Information

Publication type Journal Article ☐ Abstract ☐ Other (specify e.g. book chapter)___________________	
Country of study:	
Funding source of study:	Potential conflict of interest from funding?

**Eligibility**	Confirm eligibility for Review (Highlight the appropriate choice):<br><br>**Yes** **No**<br><br>If **No**, reason for exclusion:	
**Type of study**	☐ Case control study<br><br>☐ Observational Study– cross sectional	
**Participants**<br>(Review authors insert inclusion criteria as defined in Protocol)	Population: Gender:<br><br>Sample size calculation:	
	Do the participants meet the criteria for inclusion?	Yes ☐ No ☐<br>Unclear ☐
	Are healthy controls included in this study for comparison?	Yes ☐ No ☐<br>Unclear ☐
	Study focusing tobacco associated OSCC	Yes ☐ No ☐<br>Details:
	Histological type of OSCC<br>(Grading of OSCC)	
	Studies focusing on Genetic susceptibility/risk of development of OSCC	Yes ☐ No ☐<br>Details:

	Habit of tobacco consumption	Yes ☐ No ☐<br>Details:
	Habit of alcohol drinking	Yes ☐ No ☐<br>Details:
	Does the factor of family history of disease is considered in the study?	Yes ☐ No ☐<br>Details:
	What is the sample type used in study	Saliva ☐ Tissue ☐<br>Blood/serum/plasma ☐<br>Cell lines ☐
	Method of assessment of genetic abnormality	qPCR ☐ western blot ☐<br>Details:
	Does the study measure the odd's ratio or fold change as an outcome?	Yes ☐ No ☐<br>Details:
	Any genetic pathway explored in the study?	Yes ☐ No ☐<br>Details:
	Does the study is Homogenous<br>(if all the study samples are depicting uniform features except biological variations)	Yes ☐ No ✓☐<br>Details:
**Notes**	Key conclusions	
**Missing Data if any**		

Continuous Data (please add groups if necessary)

Outcome

1. Correlation of genetic abnormality with susceptibility/risk of development of OSCC:
   Yes ☐ No ☐

2. Which particular gene or group of gene shows high risk for development of OSCC:

3. How other confounding factors like habit of tobacco consumption, alcohol drinking and family history influences the susceptibility/risk of development of OSCC.
4. 4. Genetic mechanism if any explored in the study.

Supplementary Form 2: Risk of bias: New castle Ottawa scale

A study can be awarded a maximum of one star for each numbered item within the Selection Category. A maximum of two stars can be given for Comparability. A maximum of three stars given for outcome and Exposure categories			
Study Title:			*
Selection	1) Is the case definition as OSCC or Oral cavity cancer is adequate?	a) Yes, with Histological confirmation	
		b) Yes, with linkage to records from archival or reports	
		c)No description	

	2) Representativeness of the cases	a) Consecutive or obviously representative series of cases	
		b) Potential for selection biases or not stated	
	3) Selection of Controls	a) Healthy controls	
		b) Adjacent/contra lateral healthy area	
		c) No description	
	4) Definition of Controls	a) No history of disease	
		b) No risky habits	
Comparability	1) Comparability of cases and controls on the basis of the design or analysis	a) study controls for segregation of diseases	
		b) study controls for any additional facto r	
Exposure/Inter vention	1) Ascertainment of exposure -- Genetic assessment	a) Single gene or group of gene assessment	
		b) Odd's ratio of association or fold change mentioned	
		c) no description	
	2) Same method of gene assessment for cases and controls	Yes	
		No	
Outcome -- Susceptibility /risk of OSCC	a) Independent blind assessment		
	b) Linked to family history		
	c) Linked to risky habits		

Results in Random effects

Supplementary Figure 1: GSTM

Study or Subgroup	Experimental Events	Experimental Total	Control Events	Control Total	Weight	Odds Ratio M-H, Random, 95% CI
Deepika Shukla (2012)a	27	150	51	150	3.5%	0.43 [0.25, 0.73]
Deepika Shukla (2012)b	18	150	15	150	3.1%	1.23 [0.59, 2.54]
Deepika Shukla (2012)c	12	150	12	150	2.9%	1.00 [0.43, 2.30]
Deepika Shukla (2012)d	6	150	24	150	2.7%	0.22 [0.09, 0.55]
Edward S. Peters (2006)	287	352	345	753	3.9%	5.22 [3.85, 7.08]
Miki Sato (1999)	92	142	64	142	3.6%	2.24 [1.39, 3.61]
Mousumi Majumder (2015)a	104	310	117	348	3.9%	1.00 [0.72, 1.38]
Mousumi Majumder (2015)b	257	310	270	348	3.8%	1.40 [0.95, 2.07]
Munindra Ruwali (2011)a	86	170	340	500	3.8%	0.48 [0.34, 0.69]
Munindra Ruwali (2011)b	84	170	160	500	3.8%	2.08 [1.46, 2.96]
Munindra Ruwali (2011)c	122	170	397	500	3.8%	0.66 [0.44, 0.98]
Munindra Ruwali (2011)d	48	170	103	500	3.8%	1.52 [1.02, 2.26]
Munindra Ruwali (2011)e	111	170	285	500	3.8%	1.42 [0.99, 2.04]
Munindra Ruwali (2011)f	52	170	195	500	3.8%	0.69 [0.47, 1.00]
Munindra Ruwali (2011)g	7	170	20	500	2.8%	1.03 [0.43, 2.48]
Munindra Ruwali (2011)h	59	170	184	500	3.8%	0.91 [0.63, 1.31]
Rahmi Amtha (2009)a	32	81	72	162	3.5%	0.82 [0.47, 1.40]
Rahmi Amtha (2009)b	49	81	90	162	3.5%	1.23 [0.71, 2.11]
S.C.Buch (2018)a	146	297	111	450	3.9%	2.95 [2.16, 4.04]
S.C.Buch (2018)b	240	297	369	450	3.8%	0.92 [0.63, 1.35]
S.C.Buch (2018)c	51	297	72	450	3.8%	1.09 [0.73, 1.61]
S.C.Buch (2018)d	6	297	9	450	2.5%	1.01 [0.36, 2.87]
S.C.Buch (2018)e	243	297	395	450	3.7%	0.63 [0.42, 0.94]
S.C.Buch (2018)f	54	297	55	450	3.7%	1.60 [1.06, 2.40]
Shailendra. S. Maurya (2015)	131	300	235	750	3.9%	1.70 [1.29, 2.24]
Sreelekha T T (2001)	50	98	40	60	3.3%	0.52 [0.27, 1.01]
Tridiv Katiyar (2010)	287	529	385	1250	4.0%	2.66 [2.16, 3.28]
Zakiullah (2015)	41	200	65	151	3.6%	0.34 [0.21, 0.55]
**Total (95% CI)**		**6145**		**11426**	**100.0%**	**1.08 [0.83, 1.41]**
Total events	2702		4480			

Heterogeneity: Tau² = 0.45; Chi² = 315.77, df = 27 (P < 0.00001); I² = 91%

Test for overall effect: Z = 0.59 (P = 0.56)

Odds Ratio
M-H, Random, 95% CI

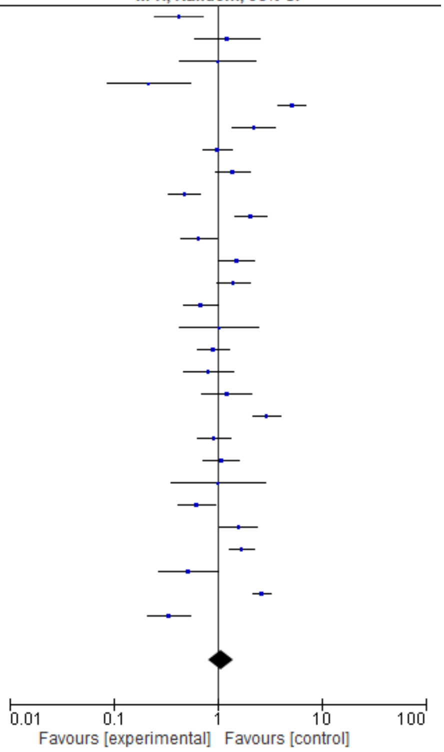

Supplementary Figure 2: UGT

Study or Subgroup	Experimental Events	Experimental Total	Control Events	Control Total	Weight	Odds Ratio M-H, Random, 95% CI
Martin Lacko (2009)	31	82	145	427	51.4%	1.18 [0.72, 1.93]
R Sharma (2010)a	11	73	29	201	21.7%	1.05 [0.50, 2.23]
R Sharma (2010)b	17	73	27	201	26.8%	1.96 [0.99, 3.85]
**Total (95% CI)**		**228**		**829**	**100.0%**	**1.32 [0.93, 1.87]**
Total events	59		201			

Heterogeneity: Tau² = 0.00; Chi² = 1.84, df = 2 (P = 0.40); I² = 0%
Test for overall effect: Z = 1.55 (P = 0.12)

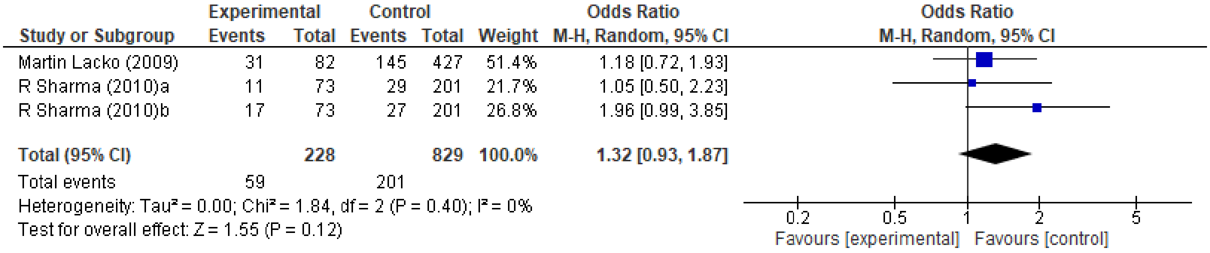

Supplementary Figure 3: Interleukin

Study or Subgroup	Experimental Events	Experimental Total	Control Events	Control Total	Weight	Odds Ratio M-H, Random, 95% CI
Ching Hao Li (2020)	38	958	31	958	4.2%	1.24 [0.76, 2.00]
Hsiu-Ting Tsai (2013)a	78	567	122	559	4.4%	0.57 [0.42, 0.78]
Hsiu-Ting Tsai (2013)b	79	567	129	559	4.4%	0.54 [0.40, 0.73]
Javier Fernández-Mateos (2019)a	43	70	31	70	3.9%	2.00 [1.02, 3.93]
Javier Fernández-Mateos (2019)b	22	70	27	70	3.9%	0.73 [0.36, 1.47]
Javier Fernández-Mateos (2019)c	5	70	12	70	3.1%	0.37 [0.12, 1.12]
Javier Fernández-Mateos (2019)d	65	70	58	70	3.1%	2.69 [0.89, 8.09]
Javier Fernández-Mateos (2019)e	5	70	12	70	3.1%	0.37 [0.12, 1.12]
Javier Fernández-Mateos (2019)f	43	70	31	70	3.9%	2.00 [1.02, 3.93]
Javier Fernández-Mateos (2019)g	27	70	39	70	3.9%	0.50 [0.25, 0.98]
Javier Fernández-Mateos (2019)h	25	70	39	70	3.9%	0.44 [0.22, 0.87]
Javier Fernández-Mateos (2019)i	33	70	23	70	3.9%	1.82 [0.92, 3.61]
Javier Fernández-Mateos (2019)j	12	70	8	70	3.4%	1.60 [0.61, 4.20]
Javier Fernández-Mateos (2019)k	58	70	62	70	3.4%	0.62 [0.24, 1.63]
Javier Fernández-Mateos (2019)l	12	70	8	70	3.4%	1.60 [0.61, 4.20]
Javier Fernández-Mateos (2019)m	25	70	39	70	3.9%	0.44 [0.22, 0.87]
Javier Fernández-Mateos (2019)n	45	70	31	70	3.9%	2.26 [1.15, 4.47]
Ming-Hsien Chien (2011)	163	240	150	240	4.4%	1.27 [0.87, 1.85]
Ming-Hsui Tsai (2005)	9	130	2	105	2.3%	3.83 [0.81, 18.13]
Prithvi Kumar Singh (2017)	116	300	197	300	4.4%	0.33 [0.24, 0.46]
Ritu Pandey (2023)a	110	166	117	151	4.2%	0.57 [0.35, 0.94]
Ritu Pandey (2023)b	72	166	52	151	4.3%	1.46 [0.93, 2.30]
Ritu Pandey (2023)c	27	166	13	151	3.8%	2.06 [1.02, 4.16]
Syed R Hussain (2016)a	168	232	95	221	4.3%	3.48 [2.35, 5.15]
Syed R Hussain (2016)b	158	232	93	221	4.4%	2.94 [2.00, 4.31]
Syed R Hussain (2016)c	121	232	48	221	4.3%	3.93 [2.61, 5.92]
**Total (95% CI)**		**4936**		**4817**	**100.0%**	**1.13 [0.81, 1.58]**
Total events	1559		1469			

Heterogeneity: $Tau^2 = 0.64$; $Chi^2 = 242.41$, df = 25 ($P < 0.00001$); $I^2 = 90\%$

Test for overall effect: Z = 0.71 (P = 0.48)

Odds Ratio M-H, Random, 95% CI

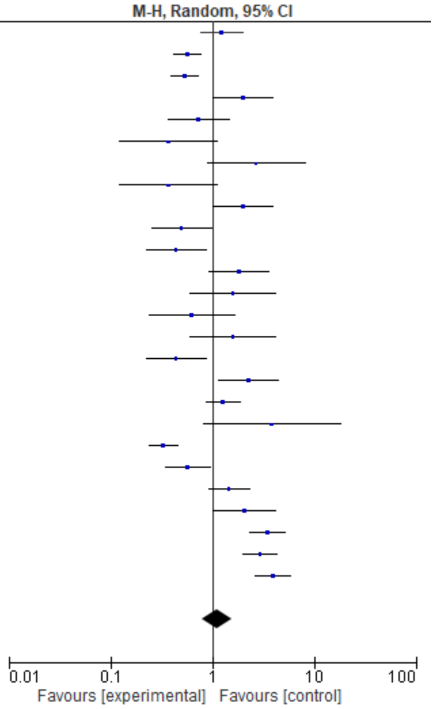

Supplementary Figure 4: VEGF

Study or Subgroup	Experimental Events	Experimental Total	Control Events	Control Total	Weight	Odds Ratio M-H, Random, 95% CI
C. Yapijakis (2007)a	64	144	39	153	18.6%	2.34 [1.43, 3.82]
C. Yapijakis (2007)b	31	144	22	153	16.1%	1.63 [0.90, 2.98]
C. Yapijakis (2007)c	58	144	42	153	18.7%	1.78 [1.10, 2.90]
Ming-Hsien Chien (2013)a	115	470	124	426	23.0%	0.79 [0.59, 1.06]
Ming-Hsien Chien (2013)b	285	470	246	426	23.6%	1.13 [0.86, 1.47]
**Total (95% CI)**		**1372**		**1311**	**100.0%**	**1.37 [0.94, 2.02]**
Total events	553		473			

Heterogeneity: $Tau^2 = 0.14$; $Chi^2 = 18.55$, df = 4 (P = 0.0010); $I^2 = 78\%$

Test for overall effect: Z = 1.63 (P = 0.10)

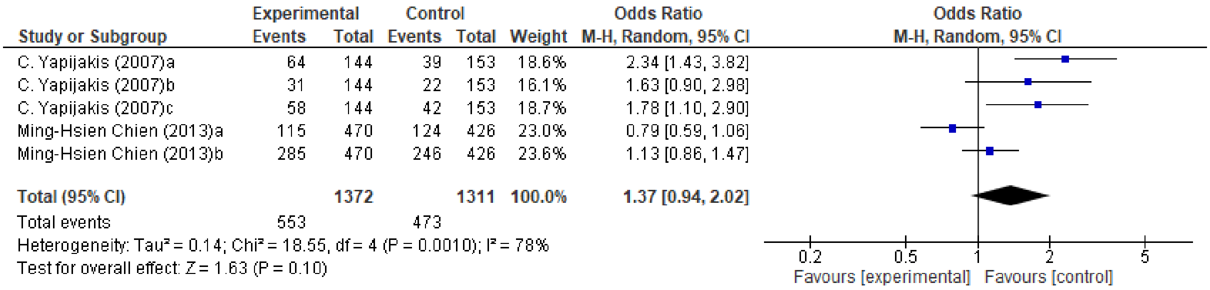

Supplementary Figure 5: MMP

Study or Subgroup	Experimental Events	Experimental Total	Control Events	Control Total	Weight	Odds Ratio M-H, Random, 95% CI
Ajay Kumar Chaudhary (2011)a	222	426	290	422	6.0%	0.50 [0.37, 0.66]
Ajay Kumar Chaudhary (2011)b	168	426	121	422	5.9%	1.62 [1.22, 2.16]
Ajay Kumar Chaudhary (2011)c	36	426	11	422	3.0%	3.45 [1.73, 6.87]
Ajay Kumar Chaudhary (2011)d	299	426	298	422	5.9%	0.98 [0.73, 1.32]
Ajay Kumar Chaudhary (2011)e	87	426	104	422	5.6%	0.78 [0.57, 1.08]
Ajay Kumar Chaudhary (2011)f	40	426	20	422	3.8%	2.08 [1.20, 3.63]
Ajay Kumar Chaudhary (2011)g	311	426	315	422	5.8%	0.92 [0.68, 1.25]
Ajay Kumar Chaudhary (2011)h	80	426	96	422	5.5%	0.79 [0.56, 1.10]
Ajay Kumar Chaudhary (2011)i	35	426	11	422	3.0%	3.34 [1.68, 6.68]
Chia-Jui Weng (2012)a	246	363	347	506	5.9%	0.96 [0.72, 1.29]
Chia-Jui Weng (2012)b	84	363	91	506	5.5%	1.37 [0.98, 1.92]
FRANCK ZINZINDOHOUÉ (2004)a	4	14	60	249	1.4%	1.26 [0.38, 4.16]
FRANCK ZINZINDOHOUÉ (2004)b	8	14	121	249	1.6%	1.41 [0.48, 4.18]
FRANCK ZINZINDOHOUÉ (2004)c	2	14	68	249	0.9%	0.44 [0.10, 2.03]
FRANCK ZINZINDOHOUÉ (2004)d	6	14	57	249	1.6%	2.53 [0.84, 7.58]
FRANCK ZINZINDOHOUÉ (2004)e	7	14	126	249	1.6%	0.98 [0.33, 2.87]
FRANCK ZINZINDOHOUÉ (2004)f	1	14	66	249	0.5%	0.21 [0.03, 1.66]
KUO-TING SUN (2016)	228	788	306	956	6.6%	0.86 [0.70, 1.06]
YI-WEN HUNG (2017)a	414	788	466	956	6.7%	1.16 [0.96, 1.41]
YI-WEN HUNG (2017)b	284	788	364	956	6.6%	0.92 [0.75, 1.11]
YI-WEN HUNG (2017)c	90	788	126	956	5.9%	0.85 [0.64, 1.13]
YI-WEN HUNG (2017)d	374	788	490	956	6.7%	0.86 [0.71, 1.04]
YI-WEN HUNG (2017)e	781	788	946	956	1.9%	1.18 [0.45, 3.11]
YI-WEN HUNG (2017)f	7	788	10	956	1.9%	0.85 [0.32, 2.24]
YI-WEN HUNG (2017)g	788	788	956	956		Not estimable
**Total (95% CI)**		**10948**		**13952**	**100.0%**	**1.05 [0.90, 1.23]**
Total events	4602		5866			

Heterogeneity: $Tau^2 = 0.09$; $Chi^2 = 88.90$, $df = 23$ ($P < 0.00001$); $I^2 = 74\%$

Test for overall effect: $Z = 0.67$ ($P = 0.50$)

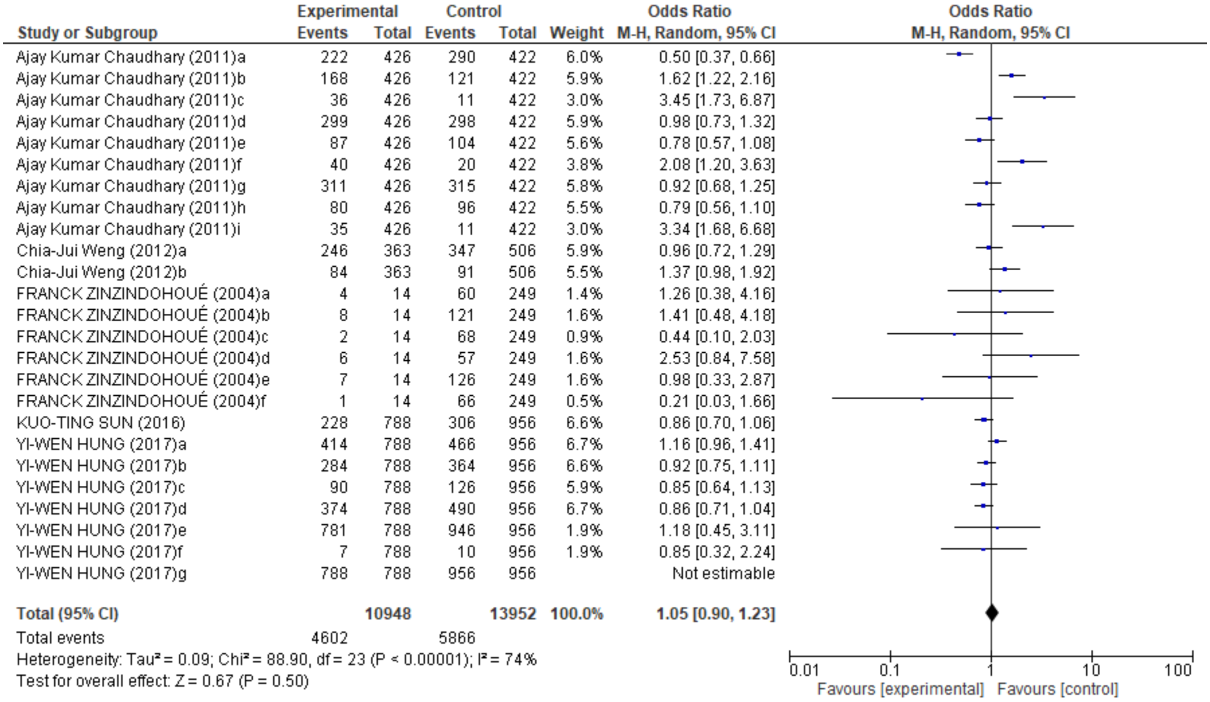

Supplementary Figure 6: Ecadherin

Study or Subgroup	Experimental Events	Experimental Total	Control Events	Control Total	Weight	Odds Ratio M-H, Random, 95% CI
Ming-Hsien Chien (2011)a	13	251	11	347	20.6%	1.67 [0.73, 3.79]
Ming-Hsien Chien (2011)b	110	251	166	347	27.0%	0.85 [0.61, 1.18]
Ming-Hsien Chien (2011)c	61	251	41	347	25.8%	2.40 [1.55, 3.70]
Ming-Hsien Chien (2011)d	106	251	65	347	26.6%	3.17 [2.20, 4.58]
**Total (95% CI)**		**1004**		**1388**	**100.0%**	**1.81 [0.90, 3.65]**
Total events	290		283			

Heterogeneity: Tau² = 0.45; Chi² = 30.71, df = 3 (P < 0.00001); I² = 90%
Test for overall effect: Z = 1.66 (P = 0.10)

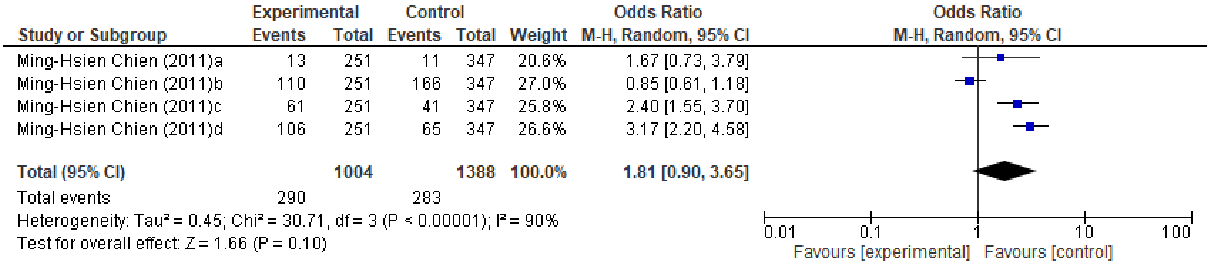

Supplementary Figure 7:CASP 8

Study or Subgroup	Experimental Events	Experimental Total	Control Events	Control Total	Weight	Odds Ratio M-H, Random, 95% CI
Chunying Li (2010)	261	295	252	1052	5.5%	24.37 [16.59, 35.79]
Costa EFD (2019)a	100	178	157	350	5.6%	1.58 [1.10, 2.27]
Costa EFD (2019)b	78	178	193	350	5.6%	0.63 [0.44, 0.91]
Costa EFD (2019)c	146	178	300	350	5.3%	0.76 [0.47, 1.24]
Costa EFD (2019)d	32	178	50	350	5.3%	1.32 [0.81, 2.14]
Costa EFD (2019)e	63	178	163	350	5.5%	0.63 [0.43, 0.91]
Costa EFD (2019)f	115	178	187	350	5.5%	1.59 [1.10, 2.31]
Costa EFD (2019)g	164	178	321	350	4.8%	1.06 [0.54, 2.06]
Costa EFD (2019)h	14	178	29	350	4.8%	0.94 [0.49, 1.84]
Costa EFD (2019)i	36	178	79	350	5.4%	0.87 [0.56, 1.35]
Costa EFD (2019)j	51	178	109	350	5.5%	0.89 [0.60, 1.32]
Costa EFD (2019)k	133	178	274	350	5.4%	0.82 [0.54, 1.25]
Costa EFD (2019)l	1	178	3	350	1.6%	0.65 [0.07, 6.33]
Kexin Chen (2014)a	92	282	365	993	5.7%	0.83 [0.63, 1.10]
Kexin Chen (2014)b	141	282	463	993	5.7%	1.14 [0.88, 1.49]
Kexin Chen (2014)c	49	282	165	993	5.6%	1.06 [0.74, 1.50]
LIANG-CHUN SHIH (2019)a	288	788	373	956	5.8%	0.90 [0.74, 1.09]
LIANG-CHUN SHIH (2019)b	47	788	67	956	5.5%	0.84 [0.57, 1.24]
LIANG-CHUN SHIH (2019)c	335	788	440	956	5.8%	0.87 [0.72, 1.05]
**Total (95% CI)**		**5641**		**11099**	**100.0%**	**1.13 [0.81, 1.58]**
Total events	2146		3990			

Heterogeneity: Tau² = 0.50; Chi² = 302.22, df = 18 (P < 0.00001); I² = 94%

Test for overall effect: Z = 0.70 (P = 0.48)

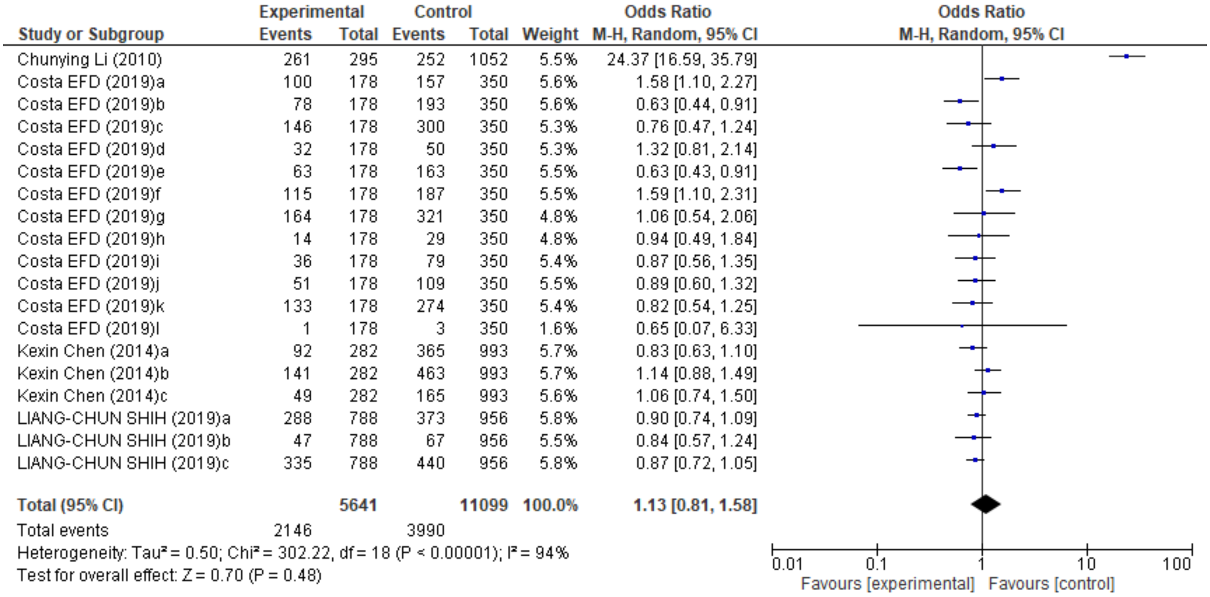

Supplementary Figure 8: XrCC 1

Study or Subgroup	Experimental Events	Experimental Total	Control Events	Control Total	Weight	Odds Ratio M-H, Random, 95% CI
Joabe dos Santos Pereira (2015)a	10	54	15	40	2.5%	0.38 [0.15, 0.97]
Joabe dos Santos Pereira (2015)b	34	54	20	40	2.8%	1.70 [0.74, 3.90]
Joabe dos Santos Pereira (2015)c	9	54	4	40	1.8%	1.80 [0.51, 6.32]
Joke Werbrouck (2008)a	10	32	69	157	2.9%	0.58 [0.26, 1.30]
Joke Werbrouck (2008)b	16	32	59	157	3.0%	1.66 [0.77, 3.57]
Joke Werbrouck (2008)c	6	32	29	157	2.4%	1.02 [0.38, 2.70]
Joke Werbrouck (2008)d	22	32	88	157	2.9%	1.73 [0.77, 3.88]
Maria Fernanda Galindez (2020)a	20	41	16	38	2.6%	1.31 [0.54, 3.18]
Maria Fernanda Galindez (2020)b	19	41	15	38	2.6%	1.32 [0.54, 3.24]
Maria Fernanda Galindez (2020)c	3	41	1	38	0.7%	2.92 [0.29, 29.37]
Mousumi Majumder (2015)a	23	150	27	150	3.6%	0.83 [0.45, 1.52]
Mousumi Majumder (2015)b	86	150	88	150	4.1%	0.95 [0.60, 1.50]
Mousumi Majumder (2015)c	81	150	92	150	4.1%	0.74 [0.47, 1.17]
Mousumi Majumder (2015)d	61	310	63	348	4.4%	1.11 [0.75, 1.64]
Mousumi Majumder (2015)e	82	310	84	348	4.5%	1.13 [0.79, 1.61]
Raktim Borkotoky (2019)a	175	310	84	348	4.6%	4.07 [2.92, 5.68]
Raktim Borkotoky (2019)b	82	310	84	348	4.5%	1.13 [0.79, 1.61]
Raktim Borkotoky (2019)c	109	310	117	348	4.6%	1.07 [0.78, 1.48]
Raktim Borkotoky (2019)d	50	152	63	190	4.2%	0.99 [0.63, 1.56]
Suparp Kietthubthew (2006)a	47	152	86	190	4.2%	0.54 [0.35, 0.85]
Suparp Kietthubthew (2006)b	52	152	41	190	4.0%	1.89 [1.17, 3.06]
Suparp Kietthubthew (2006)c	3	152	0	190	0.4%	8.92 [0.46, 174.02]
Suparp Kietthubthew (2006)d	40	106	77	164	4.0%	0.68 [0.42, 1.13]
Suparp Kietthubthew (2006)e	50	106	67	164	4.0%	1.29 [0.79, 2.11]
Suparp Kietthubthew (2006)f	16	106	20	164	3.2%	1.28 [0.63, 2.60]
Suparp Kietthubthew (2006)g	55	106	67	164	4.0%	1.56 [0.95, 2.55]
Suparp Kietthubthew (2006)h	45	106	74	164	4.0%	0.90 [0.55, 1.47]
Suparp Kietthubthew (2006)i	6	106	23	164	2.5%	0.37 [0.14, 0.94]
Suparp Kietthubthew (2006)j	83	106	140	164	3.5%	0.62 [0.33, 1.17]
Suparp Kietthubthew (2006)k	22	106	23	164	3.4%	1.61 [0.84, 3.06]
**Total (95% CI)**		**3869**		**5124**	**100.0%**	**1.11 [0.90, 1.36]**
Total events	1317		1636			

Heterogeneity: $Tau^2 = 0.21$; $Chi^2 = 107.87$, df = 29 ($P < 0.00001$); $I^2 = 73\%$

Test for overall effect: Z = 0.96 (P = 0.34)

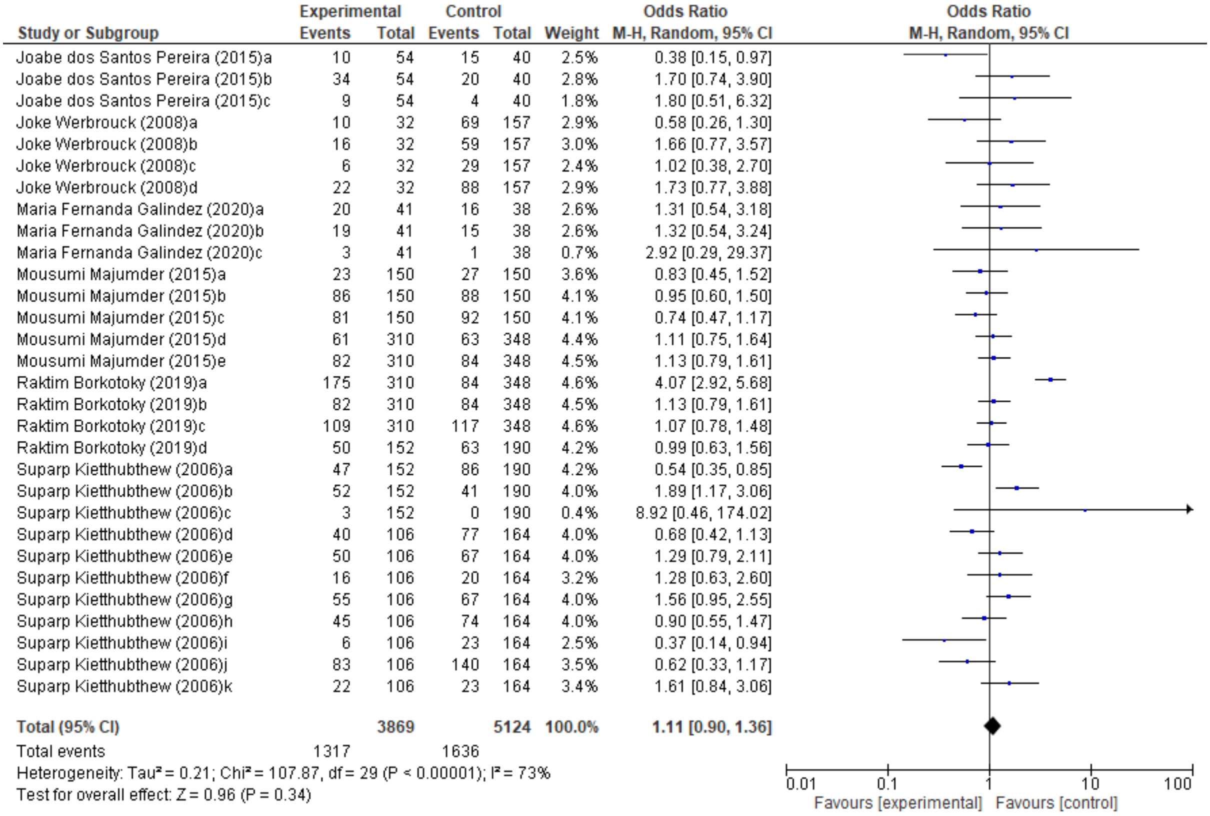

Supplementary Figure 9: MGMT

Study or Subgroup	Experimental Events	Experimental Total	Control Events	Control Total	Weight	Odds Ratio M-H, Random, 95% CI
Sung-Hsien Huang (2010)a	163	176	101	110	6.4%	1.12 [0.46, 2.71]
Sung-Hsien Huang (2010)b	0	176	1	110	0.5%	0.21 [0.01, 5.12]
Sung-Hsien Huang (2010)c	13	176	8	110	5.9%	1.02 [0.41, 2.54]
Suparp Kietthubthew (2006)a	106	106	164	164		Not estimable
Suparp Kietthubthew (2006)b	0	106	0	164		Not estimable
Suparp Kietthubthew (2006)c	0	106	0	164		Not estimable
Suparp Kietthubthew (2006)d	84	106	130	164	13.7%	1.00 [0.55, 1.82]
Suparp Kietthubthew (2006)e	21	106	33	164	13.3%	0.98 [0.53, 1.81]
Suparp Kietthubthew (2006)f	1	106	1	164	0.6%	1.55 [0.10, 25.09]
Zhengdong Zhang (2010)	93	222	567	1234	59.5%	0.85 [0.64, 1.13]
**Total (95% CI)**		**1386**		**2548**	**100.0%**	**0.91 [0.73, 1.13]**
Total events	481		1005			

Heterogeneity: Tau² = 0.00; Chi² = 1.60, df = 6 (P = 0.95); I² = 0%
Test for overall effect: Z = 0.86 (P = 0.39)

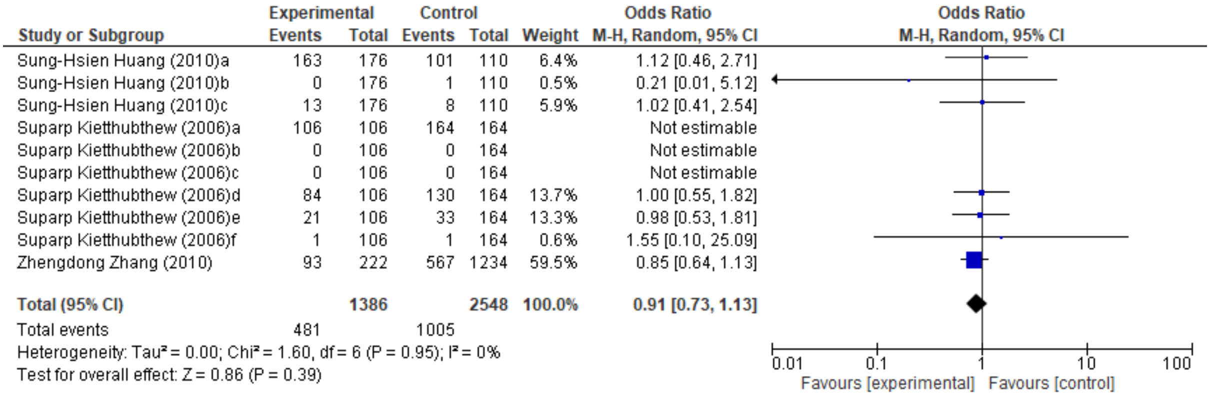

Supplementary Figure 10: P53

Study or Subgroup	Experimental Events	Experimental Total	Control Events	Control Total	Weight	Odds Ratio M-H, Random, 95% CI
Ana Maria Zarate (2017)a	41	44	7	18	6.3%	21.48 [4.76, 96.96]
Ana Maria Zarate (2017)b	23	44	14	18	6.7%	0.31 [0.09, 1.10]
Ana Maria Zarate (2017)c	2	44	9	18	6.0%	0.05 [0.01, 0.26]
DA-TIAN BAU (2007)a	112	274	109	210	7.7%	0.64 [0.45, 0.92]
DA-TIAN BAU (2007)b	125	274	105	210	7.7%	0.84 [0.59, 1.20]
Hongbing Shen (2002)a	55	105	175	333	7.6%	0.99 [0.64, 1.54]
Hongbing Shen (2002)b	41	105	134	333	7.6%	0.95 [0.61, 1.49]
Hongbing Shen (2002)c	9	105	24	333	7.3%	1.21 [0.54, 2.69]
Maria Fernanda Galindez (2020)a	8	41	14	38	7.0%	0.42 [0.15, 1.15]
Maria Fernanda Galindez (2020)b	26	41	6	38	6.9%	9.24 [3.14, 27.19]
Maria Fernanda Galindez (2020)c	7	41	5	38	6.7%	1.36 [0.39, 4.71]
Saima Saleem (2013)a	22	260	204	260	7.5%	0.03 [0.01, 0.04]
Saima Saleem (2013)b	113	260	23	260	7.6%	7.92 [4.84, 12.97]
Saima Saleem (2013)c	125	260	33	260	7.6%	6.37 [4.11, 9.88]
**Total (95% CI)**		**1898**		**2367**	**100.0%**	**1.03 [0.43, 2.43]**
Total events	709		862			

Heterogeneity: $Tau^2 = 2.49$; $Chi^2 = 381.43$, df = 13 (P < 0.00001); $I^2 = 97\%$

Test for overall effect: Z = 0.06 (P = 0.95)

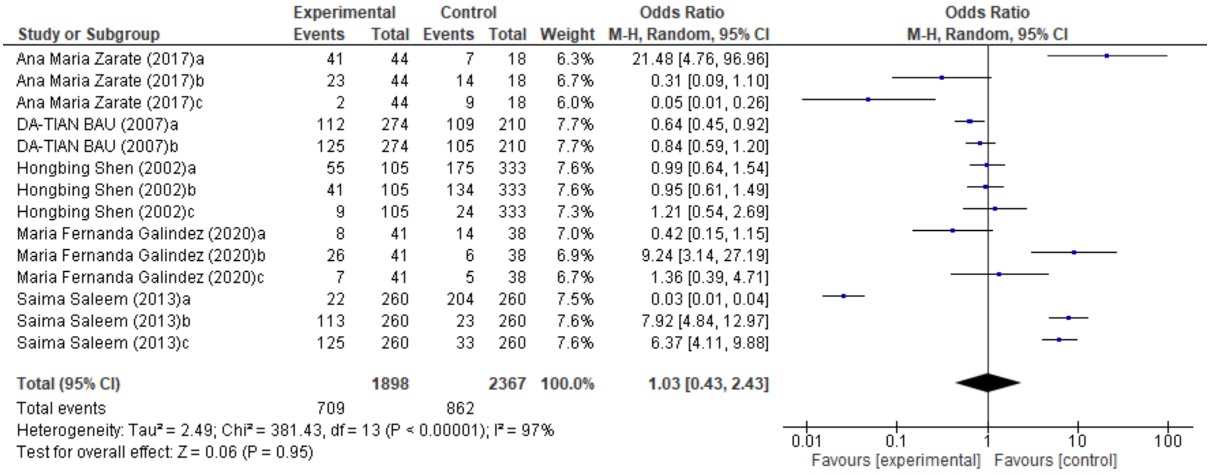

Supplementary Figure 11: Adam 1

Study or Subgroup	Experimental Events	Experimental Total	Control Events	Control Total	Weight	Odds Ratio M-H, Random, 95% CI
Yi-Tzu Chen (2023)a	32	1138	28	1199	4.2%	1.21 [0.72, 2.02]
Yi-Tzu Chen (2023)b	348	1138	398	1199	5.8%	0.89 [0.74, 1.06]
Yi-Tzu Chen (2023)c	380	1138	426	1199	5.8%	0.91 [0.77, 1.08]
Yi-Tzu Chen (2023)d	543	1138	551	1199	5.9%	1.07 [0.91, 1.26]
Yi-Tzu Chen (2023)e	222	1138	191	1199	5.7%	1.28 [1.03, 1.58]
Yi-Tzu Chen (2023)f	765	1138	742	1199	5.9%	1.26 [1.07, 1.50]
Yi-Tzu Chen (2023)g	987	1138	933	1199	5.7%	1.86 [1.50, 2.32]
Yi-Tzu Chen (2023)h	132	1138	142	1199	5.5%	0.98 [0.76, 1.26]
Yi-Tzu Chen (2023)i	2	1138	1	1199	0.6%	2.11 [0.19, 23.29]
Yi-Tzu Chen (2023)j	134	1138	143	1199	5.5%	0.99 [0.77, 1.27]
Yi-Tzu Chen (2023)k	136	1138	144	1199	5.6%	0.99 [0.77, 1.28]
Yi-Tzu Chen (2023)l	287	1138	327	1199	5.8%	0.90 [0.75, 1.08]
Yi-Tzu Chen (2023)m	21	1138	19	1199	3.7%	1.17 [0.62, 2.18]
Yi-Tzu Chen (2023)n	308	1138	346	1199	5.8%	0.91 [0.76, 1.10]
Yi-Tzu Chen (2023)o	329	1138	365	1199	5.8%	0.93 [0.78, 1.11]
Yi-Tzu Chen (2023)p	242	1138	587	1199	5.8%	0.28 [0.23, 0.34]
Yi-Tzu Chen (2023)q	267	1138	249	1199	5.8%	1.17 [0.96, 1.42]
Yi-Tzu Chen (2023)r	809	1138	836	1199	5.8%	1.07 [0.89, 1.28]
Yi-Tzu Chen (2023)s	1076	1138	1085	1199	5.2%	1.82 [1.32, 2.51]
**Total (95% CI)**		**21622**		**22781**	**100.0%**	**1.03 [0.85, 1.24]**
Total events	7020		7513			

Heterogeneity: Tau² = 0.15; Chi² = 253.51, df = 18 (P < 0.00001); I² = 93%

Test for overall effect: Z = 0.26 (P = 0.80)

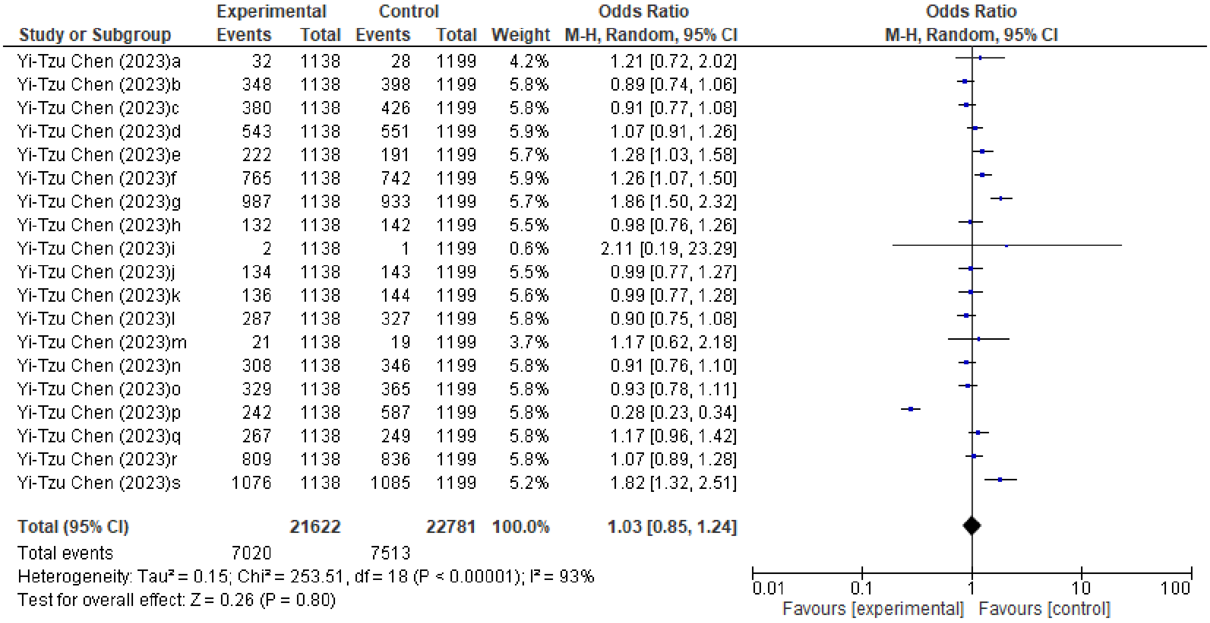

Supplementary Figure 12: LTF

Study or Subgroup	Experimental Events	Experimental Total	Control Events	Control Total	Weight	Odds Ratio M-H, Random, 95% CI
Karolina Goł abek (2022)a	25	88	30	88	11.0%	0.77 [0.40, 1.45]
Karolina Goł abek (2022)b	53	88	51	88	11.3%	1.10 [0.60, 2.00]
Karolina Goł abek (2022)c	2	88	10	88	5.6%	0.18 [0.04, 0.85]
Karolina Goł abek (2022)d	30	88	34	88	11.2%	0.82 [0.44, 1.52]
Karolina Goł abek (2022)e	55	88	44	88	11.3%	1.67 [0.91, 3.04]
Karolina Goł abek (2022)f	0	88	5	88	2.2%	0.09 [0.00, 1.58]
Karolina Goł abek (2022)g	12	88	3	88	6.7%	4.47 [1.22, 16.46]
Karolina Goł abek (2022)h	76	88	81	88	8.6%	0.55 [0.20, 1.46]
Karolina Goł abek (2022)i	12	88	17	88	9.9%	0.66 [0.29, 1.48]
Karolina Goł abek (2022)j	54	88	33	88	11.2%	2.65 [1.44, 4.86]
Karolina Goł abek (2022)k	20	88	39	88	10.9%	0.37 [0.19, 0.71]
**Total (95% CI)**		**968**		**968**	**100.0%**	**0.88 [0.55, 1.42]**
Total events	339		347			

Heterogeneity: Tau² = 0.42; Chi² = 37.61, df = 10 (P < 0.0001); I² = 73%

Test for overall effect: Z = 0.51 (P = 0.61)

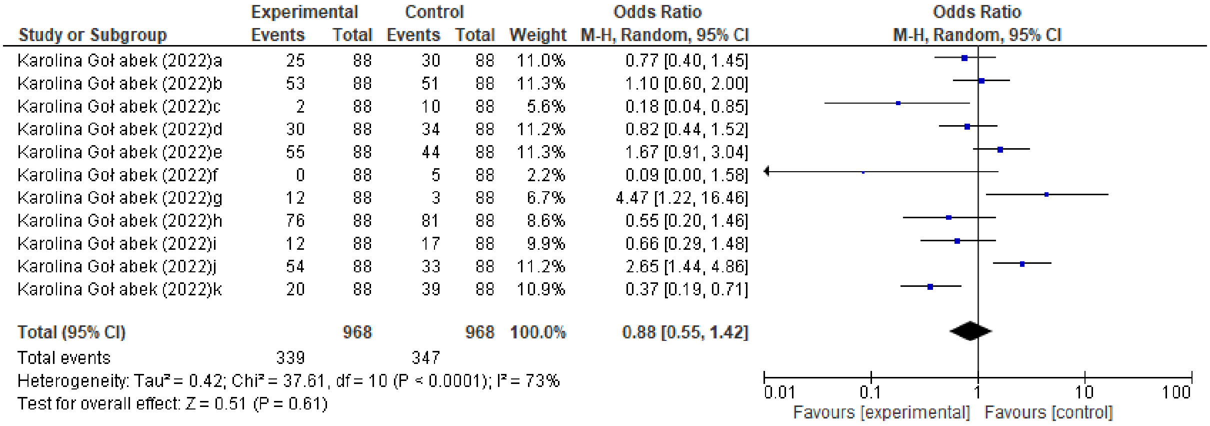

Supplementary Figure 13: MT genes

Study or Subgroup	Experimental Events	Experimental Total	Control Events	Control Total	Weight	Odds Ratio M-H, Random, 95% CI
Roberta Rezende Rosa (2021)a	9	20	11	31	7.2%	1.49 [0.47, 4.69]
Roberta Rezende Rosa (2021)b	8	20	9	31	7.0%	1.63 [0.50, 5.32]
Roberta Rezende Rosa (2021)c	5	20	1	31	3.4%	10.00 [1.07, 93.44]
Roberta Rezende Rosa (2021)d	13	20	10	31	7.0%	3.90 [1.19, 12.79]
Roberta Rezende Rosa (2021)e	7	20	20	31	7.0%	0.30 [0.09, 0.96]
Roberta Rezende Rosa (2021)f	11	20	25	31	6.7%	0.29 [0.08, 1.03]
Roberta Rezende Rosa (2021)g	6	20	14	31	7.0%	0.52 [0.16, 1.71]
Roberta Rezende Rosa (2021)h	10	20	5	31	6.5%	5.20 [1.42, 19.04]
Roberta Rezende Rosa (2021)i	16	20	19	31	6.4%	2.53 [0.68, 9.39]
Roberta Rezende Rosa (2021)j	17	20	31	39	5.8%	1.46 [0.34, 6.25]
Roberta Rezende Rosa (2021)k	13	20	25	31	6.5%	0.45 [0.12, 1.60]
Roberta Rezende Rosa (2021)l	9	20	8	31	7.0%	2.35 [0.71, 7.76]
Roberta Rezende Rosa (2021)m	1	20	1	31	2.4%	1.58 [0.09, 26.78]
Roberta Rezende Rosa (2021)n	4	20	9	31	6.3%	0.61 [0.16, 2.34]
Roberta Rezende Rosa (2021)o	15	20	16	31	6.8%	2.81 [0.82, 9.65]
Roberta Rezende Rosa (2021)p	8	20	14	31	7.2%	0.81 [0.26, 2.53]
**Total (95% CI)**		**320**		**504**	**100.0%**	**1.29 [0.79, 2.10]**
Total events	152		218			

Heterogeneity: Tau² = 0.52; Chi² = 32.80, df = 15 (P = 0.005); I² = 54%
Test for overall effect: Z = 1.01 (P = 0.31)

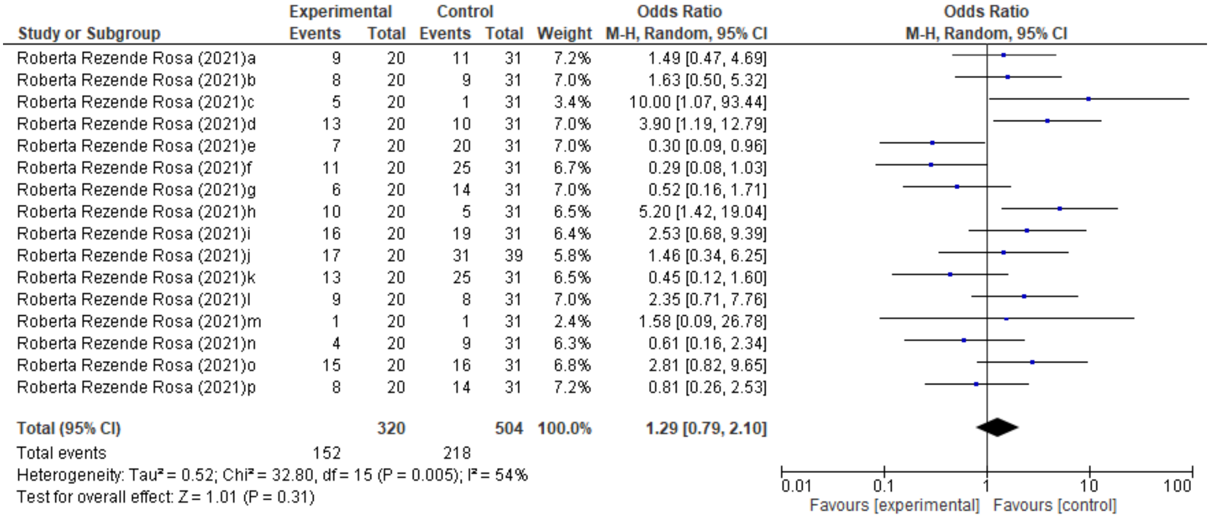

Supplementary Figure 14: XPD genes

Study or Subgroup	Experimental Events	Experimental Total	Control Events	Control Total	Weight	Odds Ratio M-H, Random, 95% CI
Joabe dos Santos Pereira (2015)a	23	54	10	40	5.9%	2.23 [0.91, 5.45]
Joabe dos Santos Pereira (2015)b	24	54	28	40	6.1%	0.34 [0.14, 0.81]
Joabe dos Santos Pereira (2015)c	7	54	2	40	3.0%	2.83 [0.56, 14.42]
Maria Fernanda Galindez (2020)a	23	41	14	38	5.9%	2.19 [0.89, 5.40]
Maria Fernanda Galindez (2020)b	31	41	7	38	4.9%	13.73 [4.63, 40.70]
Maria Fernanda Galindez (2020)c	3	41	3	38	2.9%	0.92 [0.17, 4.87]
Suparp Kietthubthew (2006)a	45	106	82	164	8.3%	0.74 [0.45, 1.21]
Suparp Kietthubthew (2006)b	52	106	62	164	8.3%	1.58 [0.97, 2.60]
Suparp Kietthubthew (2006)c	9	106	20	164	6.3%	0.67 [0.29, 1.53]
Suparp Kietthubthew (2006)d	83	106	126	164	7.7%	1.09 [0.60, 1.96]
Suparp Kietthubthew (2006)e	21	106	36	164	7.6%	0.88 [0.48, 1.61]
Suparp Kietthubthew (2006)f	1	106	2	164	1.6%	0.77 [0.07, 8.61]
Suparp Kietthubthew (2006)g	40	106	77	164	8.3%	0.68 [0.42, 1.13]
Suparp Kietthubthew (2006)h	66	106	87	164	8.3%	1.46 [0.89, 2.40]
Suparp Kietthubthew (2006)i	83	106	140	164	7.4%	0.62 [0.33, 1.17]
Suparp Kietthubthew (2006)j	23	106	24	164	7.4%	1.62 [0.86, 3.04]
**Total (95% CI)**		**1345**		**1874**	**100.0%**	**1.18 [0.84, 1.65]**
Total events	534		720			

Heterogeneity: $Tau^2 = 0.29$; $Chi^2 = 49.66$, df = 15 ($P < 0.0001$); $I^2 = 70\%$
Test for overall effect: Z = 0.97 (P = 0.33)

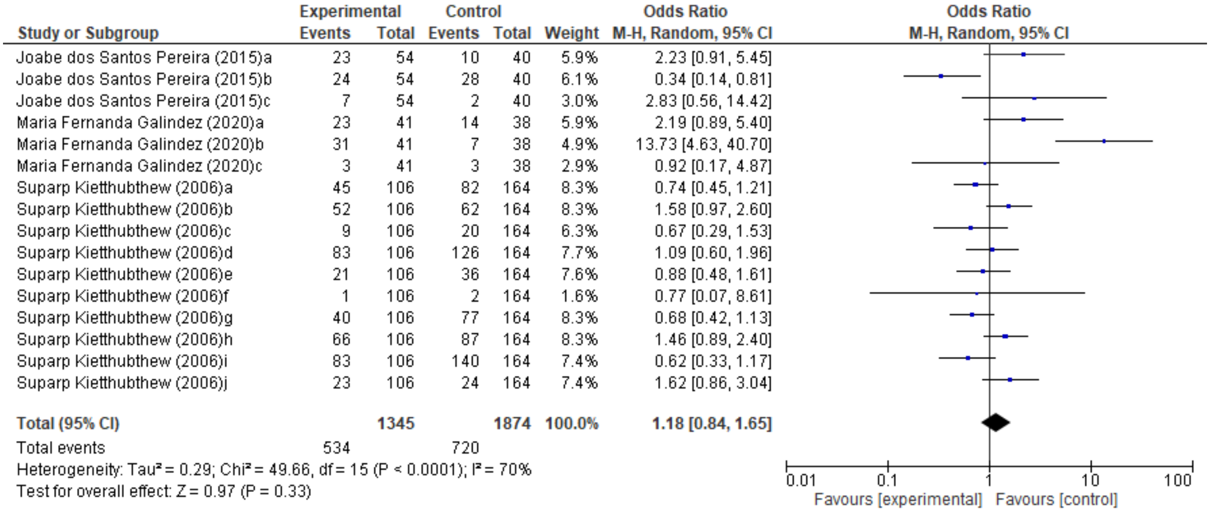

Supplementary Figure 15: NFKappa beta

Study or Subgroup	Experimental Events	Experimental Total	Control Events	Control Total	Weight	Odds Ratio M-H, Random, 95% CI
Maria Fernanda Galindez (2020)a	38	41	32	38	58.6%	2.38 [0.55, 10.26]
Maria Fernanda Galindez (2020)b	5	41	0	38	14.6%	11.60 [0.62, 217.35]
Maria Fernanda Galindez (2020)c	6	41	1	38	26.7%	6.34 [0.73, 55.38]
**Total (95% CI)**		**123**		**114**	**100.0%**	**3.90 [1.27, 11.95]**
Total events	49		33			

Heterogeneity: Tau² = 0.00; Chi² = 1.21, df = 2 (P = 0.54); I² = 0%
Test for overall effect: Z = 2.38 (P = 0.02)

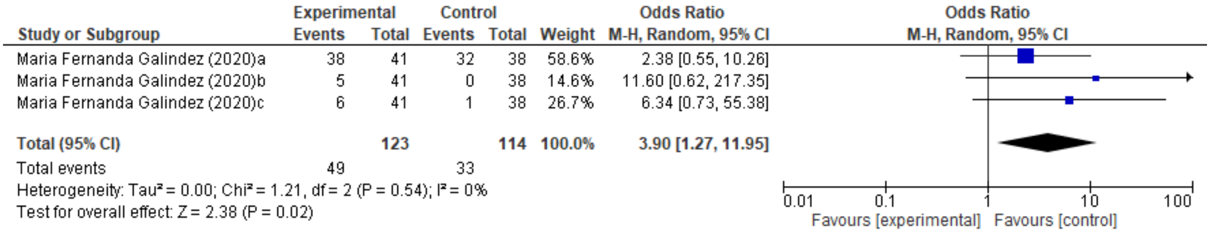

Figure 16: IGF 2

Study or Subgroup	Experimental Events	Experimental Total	Control Events	Control Total	Weight	Odds Ratio M-H, Random, 95% CI
Chia-Hsuan Chou (2020)a	831	1349	743	1198	12.6%	0.98 [0.84, 1.15]
Chia-Hsuan Chou (2020)b	466	1349	690	1198	12.6%	0.39 [0.33, 0.46]
Chia-Hsuan Chou (2020)c	52	1349	65	1198	12.1%	0.70 [0.48, 1.02]
Chia-Hsuan Chou (2020)d	518	1349	455	1198	12.6%	1.02 [0.87, 1.19]
Chia-Hsuan Chou (2020)e	779	1349	689	1198	12.6%	1.01 [0.86, 1.18]
Chia-Hsuan Chou (2020)f	499	1349	428	1198	12.6%	1.06 [0.90, 1.24]
Chia-Hsuan Chou (2020)g	711	1349	81	1198	12.4%	15.37 [11.97, 19.72]
Chia-Hsuan Chou (2020)h	570	1349	509	1198	12.6%	0.99 [0.85, 1.16]
**Total (95% CI)**		**10792**		**9584**	**100.0%**	**1.20 [0.67, 2.16]**
Total events	4426		3660			

Heterogeneity: Tau² = 0.70; Chi² = 607.85, df = 7 (P < 0.00001); I² = 99%
Test for overall effect: Z = 0.61 (P = 0.54)

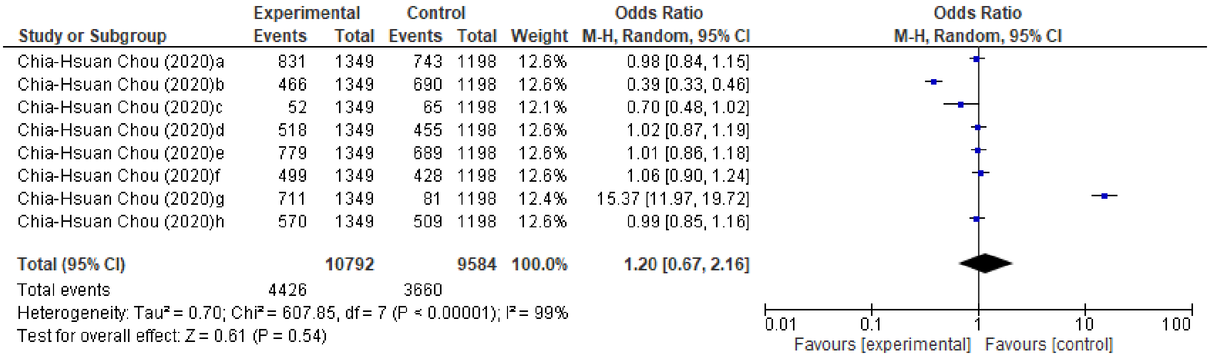

Supplementary Figure 17: LNCRNA

Study or Subgroup	Experimental Events	Experimental Total	Control Events	Control Total	Weight	Odds Ratio M-H, Random, 95% CI
C. XIN (2018)a	191	342	351	711	3.7%	1.30 [1.00, 1.68]
C. XIN (2018)b	141	342	308	711	3.7%	0.92 [0.71, 1.19]
C. XIN (2018)c	129	342	80	711	3.4%	4.78 [3.47, 6.57]
C. XIN (2018)d	200	342	409	711	3.7%	1.04 [0.80, 1.35]
C. XIN (2018)e	128	342	270	711	3.7%	0.98 [0.75, 1.28]
C. XIN (2018)f	14	342	32	711	2.0%	0.91 [0.48, 1.72]
C. XIN (2018)g	167	342	292	711	3.7%	1.37 [1.06, 1.77]
C. XIN (2018)h	148	342	280	711	3.7%	1.17 [0.90, 1.53]
C. XIN (2018)i	27	342	39	711	2.5%	1.48 [0.89, 2.46]
Jannan Ghapanchi (2020)a	110	200	133	200	3.0%	0.62 [0.41, 0.92]
Jannan Ghapanchi (2020)b	75	200	64	200	3.0%	1.27 [0.84, 1.93]
Jannan Ghapanchi (2020)c	15	200	3	200	0.8%	5.32 [1.52, 18.69]
Jannan Ghapanchi (2020)d	110	200	133	200	3.0%	0.62 [0.41, 0.92]
Jannan Ghapanchi (2020)e	90	200	67	200	3.0%	1.62 [1.08, 2.43]
Jannan Ghapanchi (2020)f	185	200	197	200	0.8%	0.19 [0.05, 0.66]
Jannan Ghapanchi (2020)g	15	200	3	200	0.8%	5.32 [1.52, 18.69]
Jannan Ghapanchi (2020)h	125	200	136	200	3.0%	0.78 [0.52, 1.18]
Jannan Ghapanchi (2020)i	75	200	64	200	3.0%	1.27 [0.84, 1.93]
Jannan Ghapanchi (2020)j	79	200	74	200	3.0%	1.11 [0.74, 1.66]
Jannan Ghapanchi (2020)k	94	200	101	200	3.1%	0.87 [0.59, 1.29]
Jannan Ghapanchi (2020)l	27	200	25	200	2.2%	1.09 [0.61, 1.96]
Jannan Ghapanchi (2020)m	79	200	74	200	3.0%	1.11 [0.74, 1.66]
Jannan Ghapanchi (2020)n	121	200	126	200	3.0%	0.90 [0.60, 1.35]
Jannan Ghapanchi (2020)o	173	200	175	200	2.2%	0.92 [0.51, 1.64]
Jannan Ghapanchi (2020)p	27	200	25	200	2.2%	1.09 [0.61, 1.96]
Jannan Ghapanchi (2020)q	106	200	99	200	3.1%	1.15 [0.78, 1.70]
Jannan Ghapanchi (2020)r	94	200	109	200	3.1%	0.74 [0.50, 1.10]
Ming-Hsien Chien (2011)a	460	1125	493	1195	4.2%	0.98 [0.83, 1.16]
Ming-Hsien Chien (2011)b	526	1125	543	1195	4.2%	1.05 [0.90, 1.24]
Ming-Hsien Chien (2011)c	139	1125	159	1195	3.8%	0.92 [0.72, 1.17]
Ming-Hsien Chien (2011)d	541	1125	595	1195	4.2%	0.93 [0.79, 1.10]
Ming-Hsien Chien (2013)a	470	1125	473	1195	4.2%	1.10 [0.93, 1.29]
Ming-Hsien Chien (2013)b	114	1125	127	1195	3.7%	0.95 [0.73, 1.24]
**Total (95% CI)**		**13428**		**17169**	**100.0%**	**1.09 [0.96, 1.23]**
Total events	4995		6059			

Heterogeneity: Tau² = 0.09; Chi² = 147.64, df = 32 (P < 0.00001); I² = 78%
Test for overall effect: Z = 1.37 (P = 0.17)

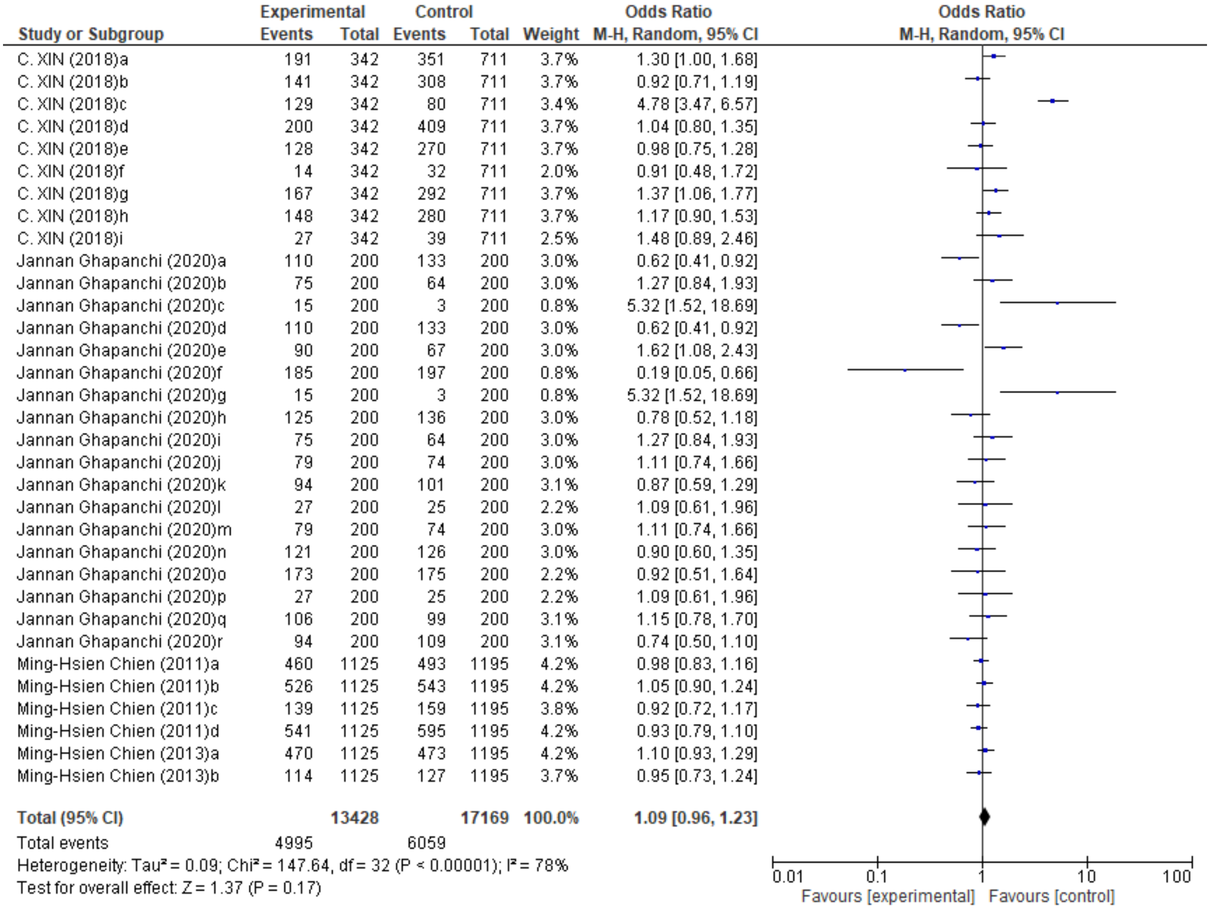

Supplementary Figure 18: TLR

Study or Subgroup	Experimental Events	Experimental Total	Control Events	Control Total	Weight	Odds Ratio M-H, Random, 95% CI
Arpita Jha (2024)a	72	120	79	115	7.3%	0.68 [0.40, 1.17]
Arpita Jha (2024)b	111	120	103	115	3.2%	1.44 [0.58, 3.55]
Arpita Jha (2024)c	42	120	24	115	6.5%	2.04 [1.14, 3.67]
Camila de Barros Gallo (2017)a	18	40	40	89	4.4%	1.00 [0.47, 2.12]
Camila de Barros Gallo (2017)b	16	40	41	89	4.3%	0.78 [0.37, 1.66]
Camila de Barros Gallo (2017)c	6	40	8	89	2.1%	1.79 [0.58, 5.54]
Camila de Barros Gallo (2017)d	10	40	12	89	3.0%	2.14 [0.84, 5.47]
Camila de Barros Gallo (2017)e	13	40	41	89	4.1%	0.56 [0.26, 1.23]
Camila de Barros Gallo (2017)f	17	40	36	89	4.3%	1.09 [0.51, 2.32]
Camila de Barros Gallo (2017)g	6	40	31	89	2.8%	0.33 [0.13, 0.87]
Camila de Barros Gallo (2017)h	21	40	34	89	4.3%	1.79 [0.84, 3.80]
Camila de Barros Gallo (2017)i	13	40	24	89	3.8%	1.30 [0.58, 2.93]
Camila de Barros Gallo (2017)j	20	40	54	89	4.3%	0.65 [0.31, 1.37]
Camila de Barros Gallo (2017)k	19	40	32	89	4.3%	1.61 [0.76, 3.43]
Camila de Barros Gallo (2017)l	1	40	3	89	0.5%	0.74 [0.07, 7.29]
Camila de Barros Gallo (2017)m	12	40	27	89	3.8%	0.98 [0.44, 2.22]
Camila de Barros Gallo (2017)n	22	40	42	89	4.4%	1.37 [0.65, 2.89]
Camila de Barros Gallo (2017)o	6	40	20	89	2.6%	0.61 [0.22, 1.66]
Camila de Barros Gallo (2017)p	29	40	58	89	3.8%	1.41 [0.62, 3.20]
Camila de Barros Gallo (2017)q	10	40	26	89	3.5%	0.81 [0.35, 1.89]
Camila de Barros Gallo (2017)r	1	40	5	89	0.6%	0.43 [0.05, 3.81]
Camila de Barros Gallo (2017)s	7	40	16	89	2.7%	0.97 [0.36, 2.58]
Camila de Barros Gallo (2017)t	21	40	37	89	4.4%	1.55 [0.73, 3.29]
Camila de Barros Gallo (2017)u	12	40	36	89	3.9%	0.63 [0.28, 1.40]
Camila de Barros Gallo (2017)v	6	40	13	89	2.4%	1.03 [0.36, 2.94]
Camila de Barros Gallo (2017)w	17	40	38	89	4.3%	0.99 [0.47, 2.11]
Camila de Barros Gallo (2017)x	17	40	38	89	4.3%	0.99 [0.47, 2.11]
**Total (95% CI)**		**1320**		**2481**	**100.0%**	**1.04 [0.88, 1.23]**
Total events	545		918			

Heterogeneity: Tau² = 0.03; Chi² = 30.35, df = 26 (P = 0.25); I² = 14%

Test for overall effect: Z = 0.45 (P = 0.66)

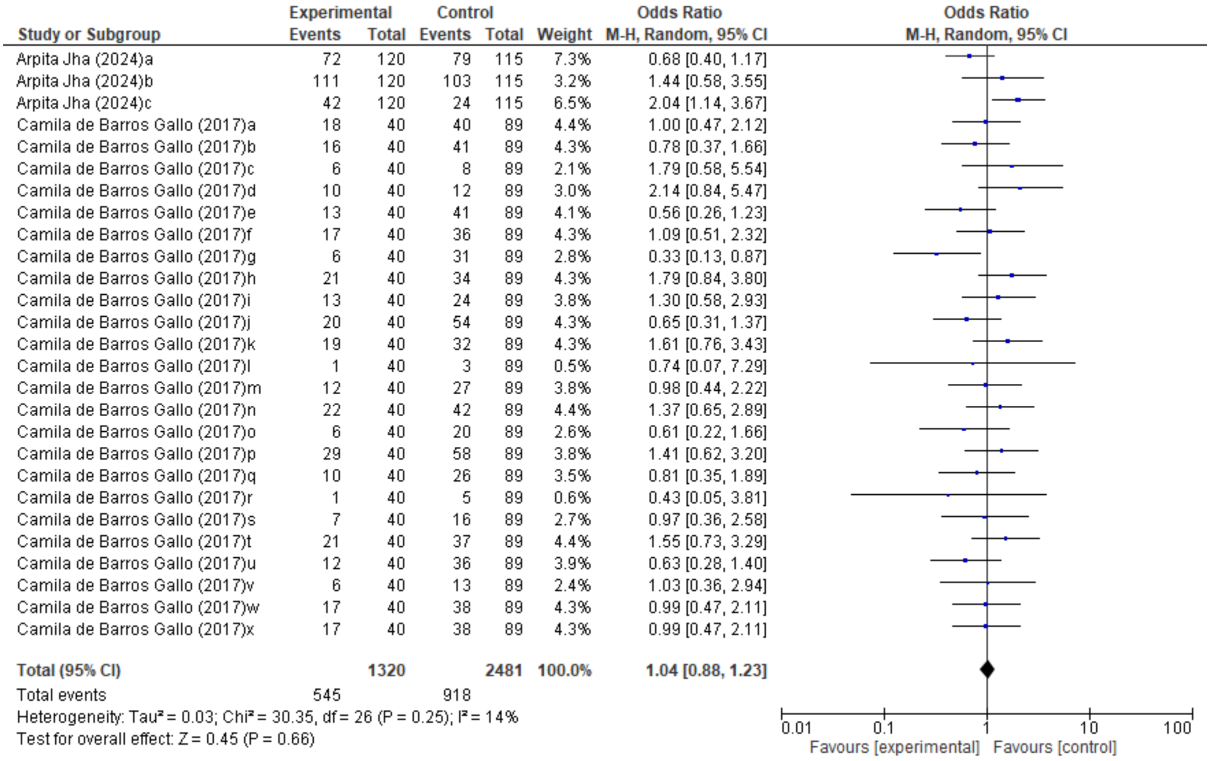

Supplementary Figure 19 :TNF

Study or Subgroup	Experimental Events	Experimental Total	Control Events	Control Total	Weight	Odds Ratio M-H, Random, 95% CI
C. MATTHIAS(2009)	17	123	15	113	10.2%	1.05 [0.50, 2.21]
Chung-Ji Liu (2005)a	175	192	120	146	10.4%	2.23 [1.16, 4.29]
Chung-Ji Liu (2005)b	16	192	24	146	10.4%	0.46 [0.24, 0.91]
Chung-Ji Liu (2005)c	4	192	10	146	8.9%	0.29 [0.09, 0.94]
Eleftherios Vairaktaris(2008)a	75	162	13	168	10.5%	10.28 [5.39, 19.59]
Eleftherios Vairaktaris(2008)b	82	162	3	168	8.9%	56.38 [17.28, 183.95]
Hsueh-Ju Lu(2022)	926	1324	832	1199	11.3%	1.03 [0.87, 1.22]
Prithvi Kumar Singh (2015)a	20	272	5	185	9.5%	2.86 [1.05, 7.75]
Prithvi Kumar Singh (2015)b	20	272	5	185	9.5%	2.86 [1.05, 7.75]
Prithvi Kumar Singh (2015)c	20	272	39	185	10.6%	0.30 [0.17, 0.53]
**Total (95% CI)**		**3163**		**2641**	**100.0%**	**1.77 [0.83, 3.78]**
Total events	1355		1066			

Heterogeneity: $Tau^2 = 1.32$; $Chi^2 = 136.17$, df = 9 ($P < 0.00001$); $I^2 = 93\%$
Test for overall effect: Z = 1.48 (P = 0.14)

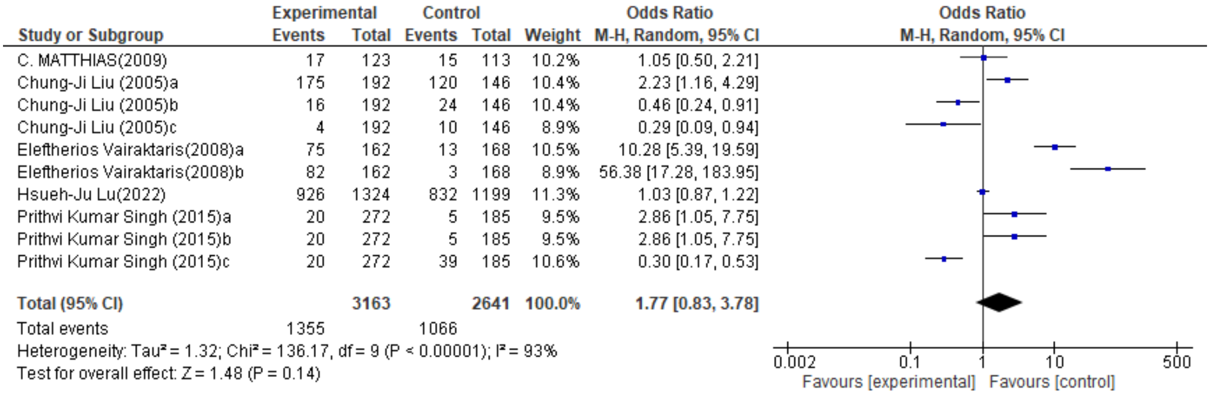

Supplementary Figure 20: P73

Study or Subgroup	Experimental Events	Experimental Total	Control Events	Control Total	Weight	Odds Ratio M-H, Random, 95% CI	Odds Ratio M-H, Random, 95% CI
Guojun Li(2003)	114	708	773	1229	33.5%	0.11 [0.09, 0.14]	
Paola Gallì(2008) a	187	283	214	295	33.2%	0.74 [0.52, 1.05]	
Paola Gallì(2008) b	96	283	81	295	33.2%	1.36 [0.95, 1.93]	
**Total (95% CI)**		**1274**		**1819**	**100.0%**	**0.48 [0.10, 2.42]**	
Total events	397		1068				

Heterogeneity: Tau² = 2.01; Chi² = 162.32, df = 2 (P < 0.00001); I² = 99%
Test for overall effect: Z = 0.89 (P = 0.37)

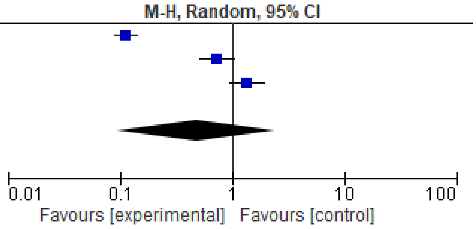

Supplementary Figure 21: MiRNA

Study or Subgroup	Experimental Events	Experimental Total	Control Events	Control Total	Weight	Odds Ratio M-H, Random, 95% CI
Divya Tandon (2017)a	37	200	16	200	2.5%	2.61 [1.40, 4.87]
Divya Tandon (2017)b	60	200	26	200	2.6%	2.87 [1.72, 4.78]
Divya Tandon (2017)c	83	200	36	200	2.7%	3.23 [2.05, 5.11]
Divya Tandon (2017)d	140	200	174	200	2.6%	0.35 [0.21, 0.58]
Divya Tandon (2017)e	37	200	16	200	2.5%	2.61 [1.40, 4.87]
Divya Tandon (2017)f	23	200	10	200	2.2%	2.47 [1.14, 5.33]
Divya Tandon (2017)g	60	200	26	200	2.6%	2.87 [1.72, 4.78]
Enjiao Zhang(2017)a	169	340	155	340	2.9%	1.18 [0.87, 1.59]
Enjiao Zhang(2017)b	27	340	19	340	2.5%	1.46 [0.79, 2.67]
Enjiao Zhang(2017)c	31	340	12	340	2.4%	2.74 [1.38, 5.44]
Limin Miao(2015)a	460	462	1549	1552	1.0%	0.45 [0.07, 2.67]
Limin Miao(2015)b	278	461	798	1548	3.0%	1.43 [1.16, 1.76]
Limin Miao(2015)c	112	461	292	1548	3.0%	1.38 [1.08, 1.77]
Limin Miao(2015)d	140	461	631	1548	3.0%	0.63 [0.51, 0.79]
Owen M. Wilkins(2017)	50	712	264	2407	2.9%	0.61 [0.45, 0.84]
PS Sushma(2015)a	68	100	81	102	2.4%	0.55 [0.29, 1.04]
PS Sushma(2015)b	10	100	15	102	2.1%	0.64 [0.27, 1.51]
PS Sushma(2015)c	22	100	6	102	1.9%	4.51 [1.74, 11.68]
PS Sushma(2015)d	32	100	21	102	2.4%	1.82 [0.96, 3.44]
PS Sushma(2015)e	78	100	96	102	1.9%	0.22 [0.09, 0.57]
PS Sushma(2015)f	72	100	90	102	2.3%	0.34 [0.16, 0.72]
PS Sushma(2015)g	22	100	12	102	2.2%	2.12 [0.98, 4.55]
PS Sushma(2015)h	6	100	0	102	0.5%	14.10 [0.78, 253.71]
PS Sushma(2015)i	26	100	12	102	2.3%	2.64 [1.24, 5.58]
PS Sushma(2015)j	94	100	102	102	0.5%	0.07 [0.00, 1.28]
Roshni Roy(2013)a	218	451	242	452	3.0%	0.81 [0.63, 1.05]
Roshni Roy(2013)b	248	451	286	452	3.0%	0.71 [0.54, 0.93]
Shikha Tiwari(2023)a	124	215	127	215	2.8%	0.94 [0.64, 1.39]
Shikha Tiwari(2023)b	90	215	88	215	2.8%	1.04 [0.71, 1.53]
Shikha Tiwari(2023)c	78	215	62	215	2.8%	1.40 [0.94, 2.11]
Shikha Tiwari(2023)d	91	215	28	215	2.7%	4.90 [3.03, 7.93]
Shikha Tiwari(2023)e	43	215	23	215	2.6%	2.09 [1.21, 3.60]
Shikha Tiwari(2023)f	78	215	62	215	2.8%	1.40 [0.94, 2.11]
Shikha Tiwari(2023)g	137	215	152	215	2.8%	0.73 [0.49, 1.09]
Shikha Tiwari(2023)h	171	215	191	215	2.6%	0.49 [0.29, 0.84]
Yin-Hung Chu(2012)a	43	215	23	215	2.6%	2.09 [1.21, 3.60]
Yin-Hung Chu(2012)b	12	470	3	425	1.5%	3.69 [1.03, 13.15]
Yin-Hung Chu(2012)c	107	470	17	425	2.6%	7.07 [4.16, 12.03]
Y-Y Hou(2015)a	110	470	31	425	2.8%	3.88 [2.54, 5.93]
Y-Y Hou(2015)b	111	155	152	204	2.7%	0.86 [0.54, 1.38]
Y-Y Hou(2015)c	39	155	51	204	2.7%	1.01 [0.62, 1.63]
**Total (95% CI)**		**10534**		**16565**	**100.0%**	**1.37 [1.10, 1.70]**
Total events	3837		5997			

Heterogeneity: $Tau^2 = 0.40$; $Chi^2 = 353.09$, df = 40 ($P < 0.00001$); $I^2 = 89\%$
Test for overall effect: Z = 2.84 (P = 0.004)

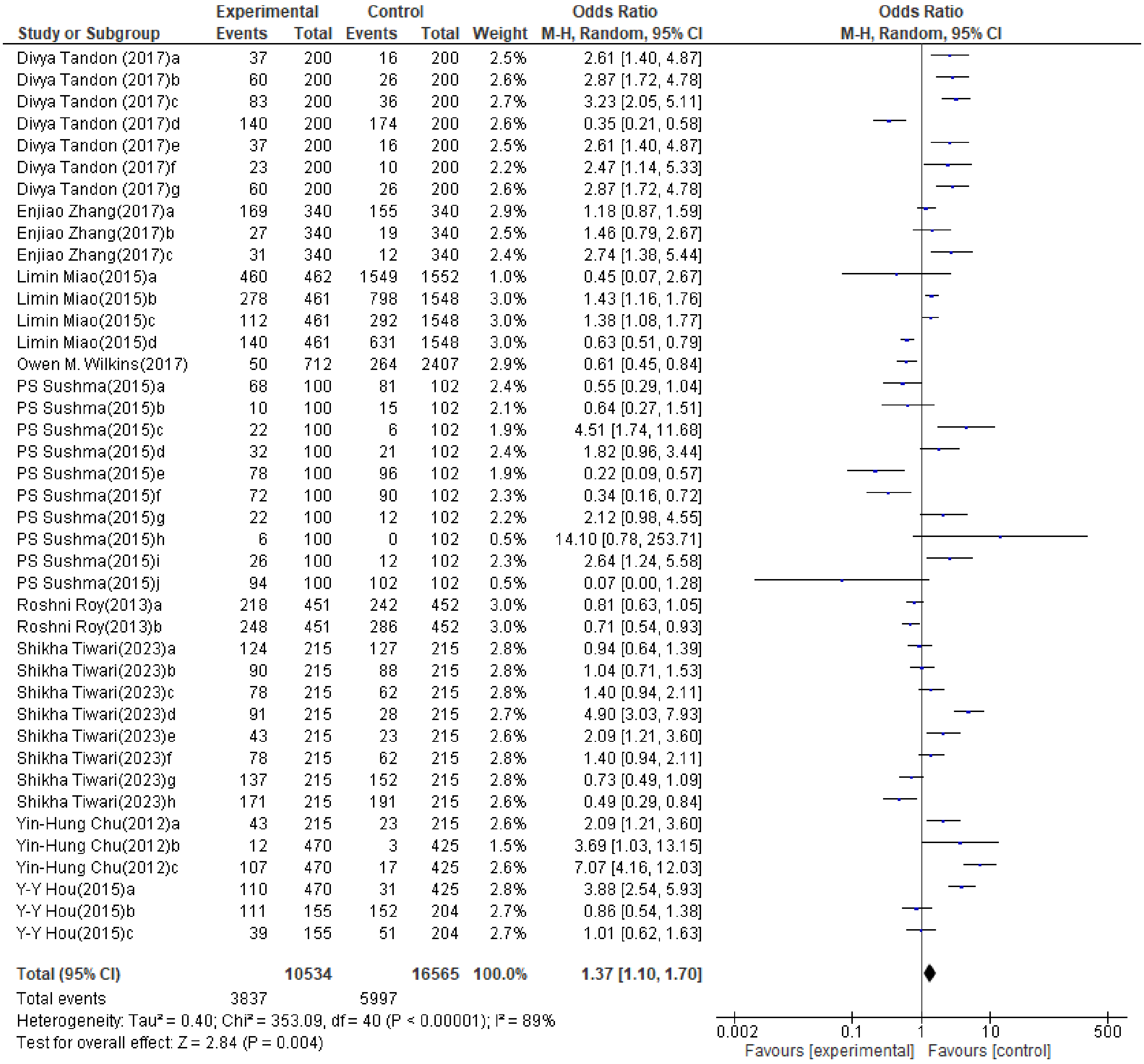

Supplementary Figure 22 : CYP

Study or Subgroup	Experimental Events	Experimental Total	Control Events	Control Total	Weight	Odds Ratio M-H, Random, 95% CI
Deepika Shukla(2012)a	60	150	72	150	3.9%	0.72 [0.46, 1.14]
Deepika Shukla(2012)b	6	150	45	150	3.4%	0.10 [0.04, 0.24]
In-Ho Cha(2007)a	22	72	17	221	3.6%	5.28 [2.61, 10.68]
In-Ho Cha(2007)b	10	72	4	221	2.9%	8.75 [2.65, 28.86]
In-Ho Cha(2007)c	10	11	13	221	1.8%	160.00 [19.00, 1347.22]
Katarina Zeljic(2012)	65	110	90	122	3.8%	0.51 [0.30, 0.89]
Lakshmi Balaji(2011)Lakshmi Balaji(2011)	6	151	7	125	3.0%	0.70 [0.23, 2.13]
Miki Sato(1999)	31	142	15	142	3.7%	2.36 [1.21, 4.61]
Rahmi Amtha(2009)a	10	73	34	201	3.5%	0.78 [0.36, 1.67]
Rahmi Amtha(2009)b	2	73	0	201	1.1%	14.09 [0.67, 297.04]
R Sharma(2010)a	6	73	19	201	3.3%	0.86 [0.33, 2.24]
R Sharma(2010)b	45	81	77	162	3.8%	1.38 [0.81, 2.36]
R Sharma(2010)c	36	81	85	162	3.8%	0.72 [0.42, 1.24]
S. Liu(2000)	106	171	104	399	4.0%	4.63 [3.16, 6.77]
Seram Anil Singh(2015)a	25	63	83	230	3.8%	1.17 [0.66, 2.06]
Seram Anil Singh(2015)b	12	63	22	230	3.5%	2.22 [1.03, 4.79]
Seram Anil Singh(2015)c	37	63	105	230	3.8%	1.69 [0.96, 2.98]
Shailendra. S. Maurya (2015)a	30	300	44	750	3.9%	1.78 [1.10, 2.90]
Shailendra. S. Maurya (2015)b	30	300	39	750	3.9%	2.03 [1.23, 3.33]
Shailendra. S. Maurya (2015)c	30	300	19	750	3.7%	4.27 [2.37, 7.72]
Shailendra. S. Maurya (2015)d	29	300	35	750	3.8%	2.19 [1.31, 3.65]
Shou-Yen Kao(2002)a	8	106	66	146	3.5%	0.10 [0.04, 0.22]
Shou-Yen Kao(2002)b	84	106	78	146	3.8%	3.33 [1.88, 5.89]
Shou-Yen Kao(2002)c	8	106	2	146	2.4%	5.88 [1.22, 28.27]
Shou-Yen Kao(2002)d	40	106	53	146	3.8%	1.06 [0.63, 1.78]
Shou-Yen Kao(2002)e	52	106	79	146	3.8%	0.82 [0.49, 1.35]
Shou-Yen Kao(2002)f	14	106	14	146	3.5%	1.43 [0.65, 3.15]
Sreelekha T T(2001)	50	98	10	60	3.5%	5.21 [2.37, 11.43]
Zeki Topcu(2002)	16	286	26	135	3.7%	0.25 [0.13, 0.48]
**Total (95% CI)**		**3819**		**7439**	**100.0%**	**1.50 [1.03, 2.19]**
Total events	880		1257			

Heterogeneity: Tau² = 0.90; Chi² = 270.29, df = 28 (P < 0.00001); I² = 90%
Test for overall effect: Z = 2.09 (P = 0.04)

Odds Ratio
M-H, Random, 95% CI

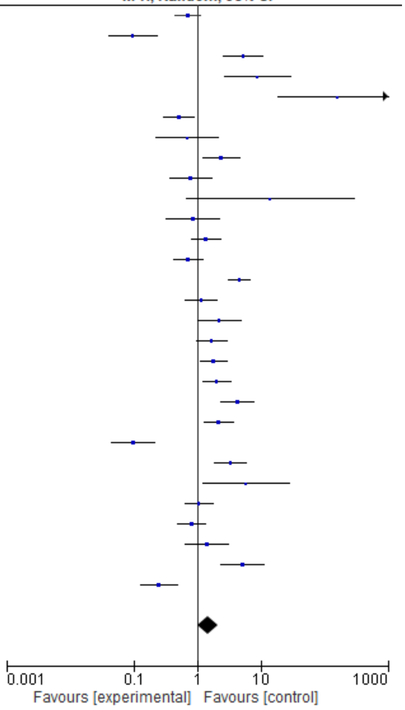

Supplementary Figure 23: MTHFR

Study or Subgroup	Experimental Events	Experimental Total	Control Events	Control Total	Weight	Odds Ratio M-H, Random, 95% CI
Ana Livia Silva Galbiatti(2012)	81	129	49	531	7.5%	16.60 [10.45, 26.36]
Ana S. Neumann(2005)	139	216	387	545	7.8%	0.74 [0.53, 1.03]
Andressa Barbosa(2015)	231	404	213	401	7.8%	1.18 [0.89, 1.56]
Ebrahim Miri-Moghaddam(2016)a	34	57	47	62	6.8%	0.47 [0.21, 1.04]
Ebrahim Miri-Moghaddam(2016)b	21	57	14	62	6.7%	2.00 [0.90, 4.46]
Ebrahim Miri-Moghaddam(2016)c	2	57	1	62	3.0%	2.22 [0.20, 25.14]
Ebrahim Miri-Moghaddam(2016)d	30	57	34	62	7.0%	0.92 [0.44, 1.88]
Ebrahim Miri-Moghaddam(2016)e	26	57	26	62	6.9%	1.16 [0.56, 2.40]
Ebrahim Miri-Moghaddam(2016)f	1	57	2	62	3.0%	0.54 [0.05, 6.07]
R. Sailasree(2010)a	37	130	46	139	7.4%	0.80 [0.48, 1.35]
R. Sailasree(2010)b	74	130	59	139	7.5%	1.79 [1.10, 2.91]
R. Sailasree(2010)d	19	130	34	139	7.2%	0.53 [0.28, 0.98]
R. Sailasree(2010)e	92	130	108	139	7.4%	0.69 [0.40, 1.20]
R. Sailasree(2010)f	8	130	29	139	6.7%	0.25 [0.11, 0.57]
R. Sailasree(2010)g	92	130	108	139	7.4%	0.69 [0.40, 1.20]
**Total (95% CI)**		**1871**		**2683**	**100.0%**	**1.04 [0.62, 1.76]**
Total events	887		1157			

Heterogeneity: $Tau^2 = 0.90$; $Chi^2 = 173.41$, df = 14 (P < 0.00001); $I^2 = 92\%$

Test for overall effect: Z = 0.15 (P = 0.88)

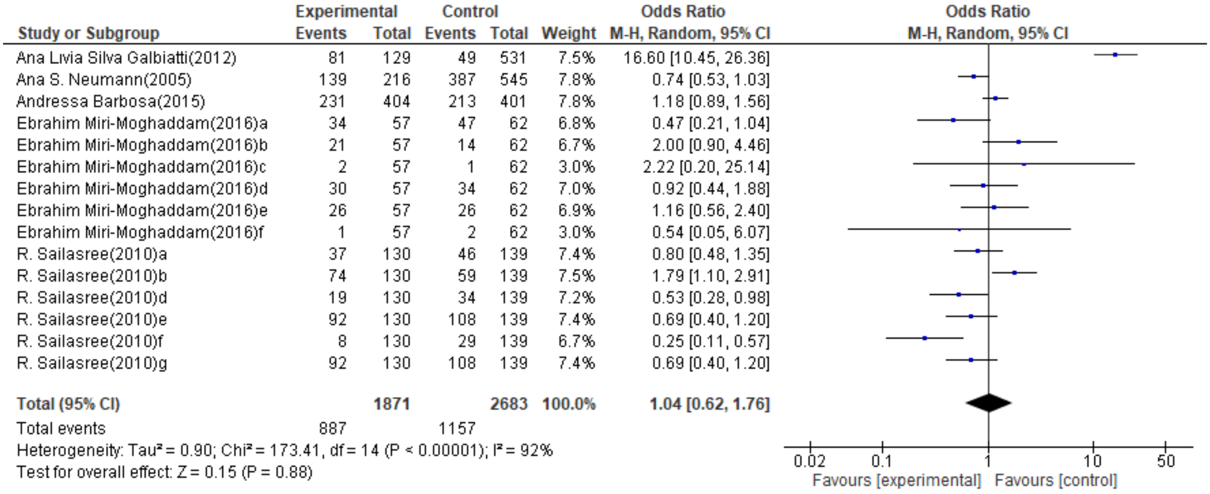

Supplementary Figure 24: Wisp

Study or Subgroup	Experimental Events	Experimental Total	Control Events	Control Total	Weight	Odds Ratio M-H, Random, 95% CI
Hon-Kit Lau (2017)a	707	900	926	1200	5.3%	1.08 [0.88, 1.34]
Hon-Kit Lau (2017)b	180	900	253	1200	5.0%	0.94 [0.76, 1.16]
Hon-Kit Lau (2017)c	13	900	21	1200	0.5%	0.82 [0.41, 1.65]
Hon-Kit Lau (2017)d	662	900	877	1200	6.1%	1.02 [0.84, 1.25]
Hon-Kit Lau (2017)e	222	900	298	1200	5.8%	0.99 [0.81, 1.21]
Hon-Kit Lau (2017)f	16	900	25	1200	0.6%	0.85 [0.45, 1.60]
Hon-Kit Lau (2017)g	263	900	337	1200	6.3%	1.06 [0.87, 1.28]
Hon-Kit Lau (2017)h	435	900	573	1200	7.7%	1.02 [0.86, 1.22]
Hon-Kit Lau (2017)i	202	900	290	1200	5.5%	0.91 [0.74, 1.11]
Hon-Kit Lau (2017)j	233	900	330	1200	6.0%	0.92 [0.76, 1.12]
Hon-Kit Lau (2017)k	435	900	581	1200	7.7%	1.00 [0.84, 1.18]
Hon-Kit Lau (2017)l	232	900	289	1200	5.8%	1.09 [0.90, 1.34]
Hon-Kit Lau (2017)m	302	900	439	1200	7.0%	0.88 [0.73, 1.05]
Hon-Kit Lau (2017)n	433	900	583	1200	7.7%	0.98 [0.83, 1.17]
Hon-Kit Lau (2017)o	155	900	178	1200	4.2%	1.19 [0.94, 1.51]
Hon-Kit Lau (2017)p	395	900	503	1200	7.6%	1.08 [0.91, 1.29]
Hon-Kit Lau (2017)q	391	900	560	1200	7.7%	0.88 [0.74, 1.04]
Hon-Kit Lau (2017)r	114	900	137	1200	3.3%	1.13 [0.86, 1.47]
**Total (95% CI)**		**16200**		**21600**	**100.0%**	**1.00 [0.95, 1.05]**
Total events	5390		7200			

Heterogeneity: Tau² = 0.00; Chi² = 12.30, df = 17 (P = 0.78); I² = 0%

Test for overall effect: Z = 0.13 (P = 0.89)

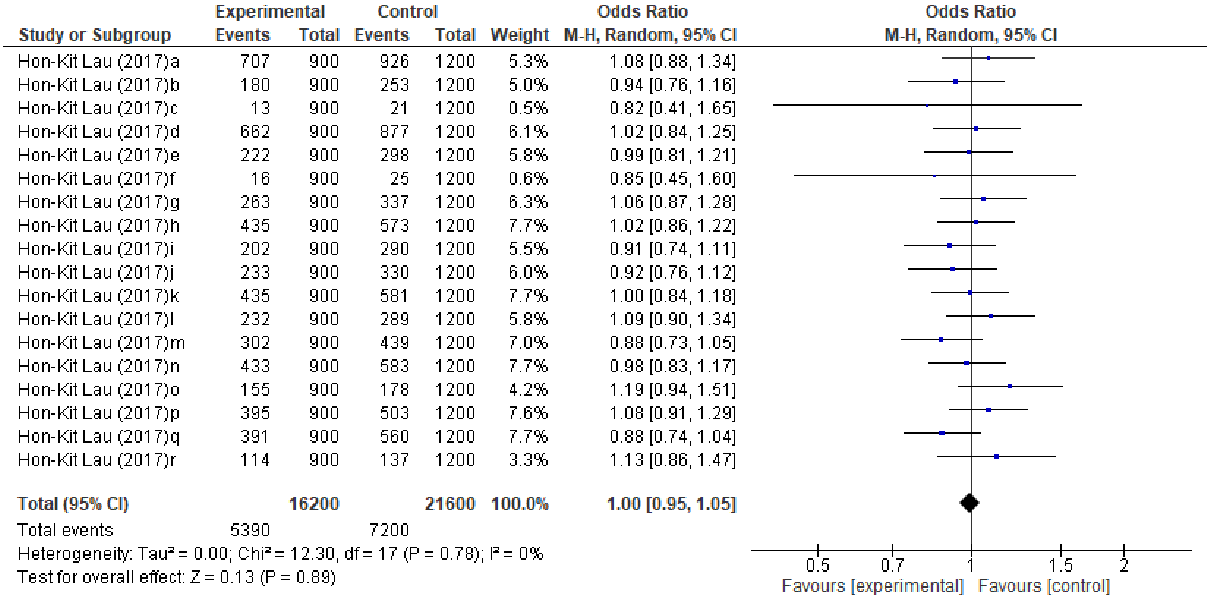

Supplementary Figure 25: COX 2

Study or Subgroup	Experimental Events	Experimental Total	Control Events	Control Total	Weight	Odds Ratio M-H, Random, 95% CI
Canan Cacina (2018)a	11	114	35	165	9.7%	0.40 [0.19, 0.82]
Canan Cacina (2018)b	82	114	98	165	10.0%	1.75 [1.05, 2.93]
Yuming Niu (2014)a	44	169	103	222	10.1%	0.41 [0.26, 0.63]
Yuming Niu (2014)b	80	169	103	542	10.2%	3.83 [2.65, 5.55]
Yuming Niu (2014)c	45	169	103	271	10.1%	0.59 [0.39, 0.90]
Yuming Niu (2014)d	125	169	103	813	10.1%	19.58 [13.11, 29.24]
Yuming Niu (2014)e	118	168	103	691	10.1%	13.47 [9.11, 19.93]
Yuming Niu (2014)f	45	168	103	316	10.1%	0.76 [0.50, 1.15]
Yuming Niu (2014)g	5	168	25	103	9.3%	0.10 [0.04, 0.26]
Yuming Niu (2014)h	50	168	103	341	10.1%	0.98 [0.65, 1.47]
**Total (95% CI)**		**1576**		**3629**	**100.0%**	**1.32 [0.50, 3.44]**
Total events	605		879			

Heterogeneity: $Tau^2 = 2.32$; $Chi^2 = 400.84$, df = 9 ($P < 0.00001$); $I^2 = 98\%$

Test for overall effect: Z = 0.56 (P = 0.57)

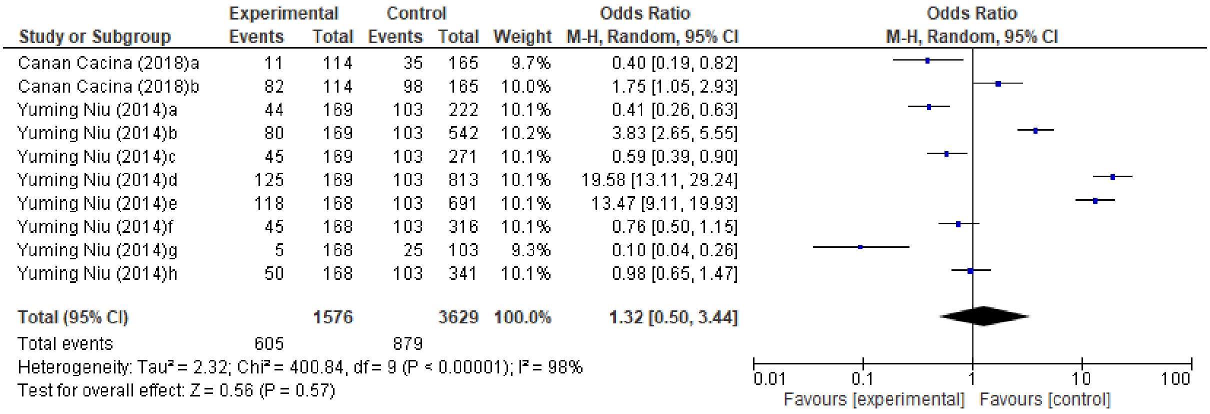

Supplementary Figure 26: Survivin

Study or Subgroup	Experimental Events	Experimental Total	Control Events	Control Total	Weight	Odds Ratio M-H, Random, 95% CI
Canan Cacina (2018)a	56	61	118	133	6.2%	1.42 [0.49, 4.11]
Canan Cacina (2018)b	35	61	65	133	18.6%	1.41 [0.76, 2.59]
Marija Kostić (2013)a	39	88	45	111	21.7%	1.17 [0.66, 2.06]
Marija Kostić (2013)b	36	88	53	111	21.7%	0.76 [0.43, 1.33]
Marija Kostić (2013)c	13	88	13	111	10.2%	1.31 [0.57, 2.98]
Marija Kostić (2013)d	49	88	66	111	21.7%	0.86 [0.49, 1.51]
**Total (95% CI)**		**474**		**710**	**100.0%**	**1.05 [0.81, 1.37]**
Total events	228		360			

Heterogeneity: Tau² = 0.00; Chi² = 3.39, df = 5 (P = 0.64); I² = 0%

Test for overall effect: Z = 0.39 (P = 0.70)

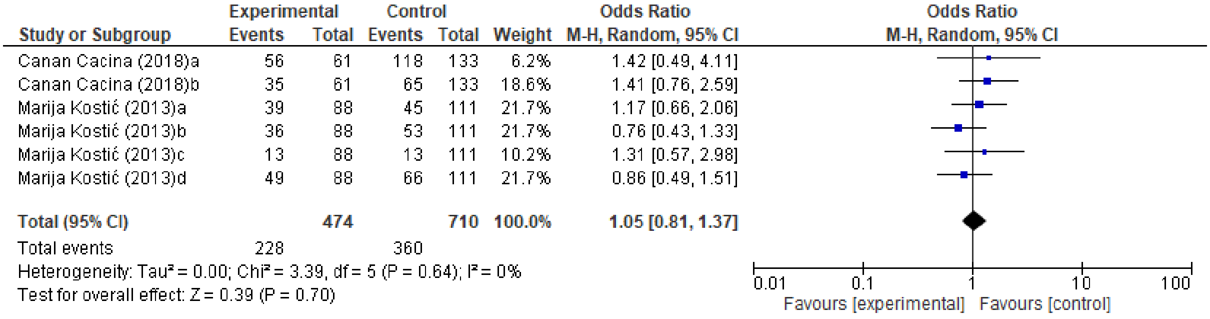

Supplementary Figure 27: RAD 5

Study or Subgroup	Experimental Events	Experimental Total	Control Events	Control Total	Weight	Odds Ratio M-H, Random, 95% CI
Jiachun Lu (2007)a	24	716	240	719	24.8%	0.07 [0.04, 0.11]
Jiachun Lu (2007)b	180	716	335	719	25.4%	0.38 [0.31, 0.48]
Joke Werbrouck (2008)a	31	32	134	157	15.5%	5.32 [0.69, 40.92]
Joke Werbrouck (2008)b	0	32	3	157	10.7%	0.68 [0.03, 13.47]
Joke Werbrouck (2008)c	1	32	0	157	9.7%	15.00 [0.60, 376.70]
Joke Werbrouck (2008)d	1	32	3	157	14.0%	1.66 [0.17, 16.45]
**Total (95% CI)**		**1560**		**2066**	**100.0%**	**0.70 [0.20, 2.52]**
Total events	237		715			

Heterogeneity: Tau² = 1.66; Chi² = 64.92, df = 5 (P < 0.00001); I² = 92%

Test for overall effect: Z = 0.54 (P = 0.59)

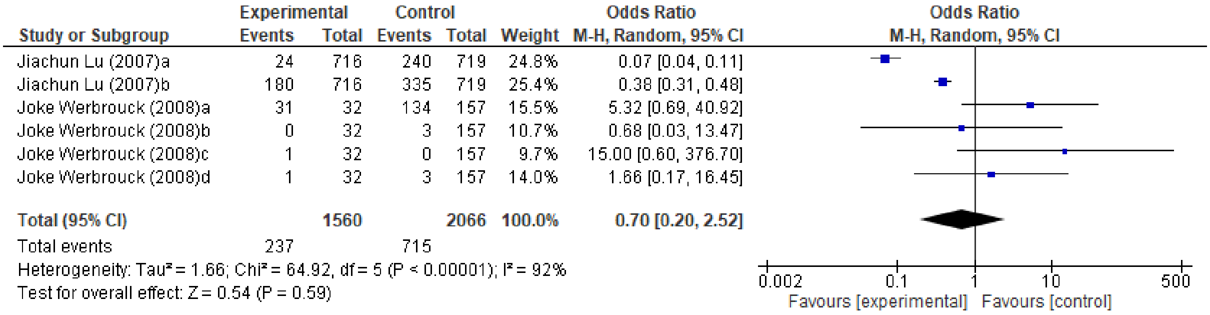

Supplementary Figure 28: ADH1C

Study or Subgroup	Experimental Events	Experimental Total	Control Events	Control Total	Weight	Odds Ratio M-H, Random, 95% CI
Edward S. Peters (2005)	242	256	273	599	19.9%	20.64 [11.76, 36.22]
Miroslav Brocic (2011)a	24	123	72	177	20.0%	0.35 [0.21, 0.61]
Miroslav Brocic (2011)b	20	123	25	177	19.7%	1.18 [0.62, 2.24]
Sheng Wei, (2010)a	309	327	1007	1129	20.1%	2.08 [1.25, 3.47]
Sheng Wei, (2010)b	286	327	931	1129	20.4%	1.48 [1.03, 2.13]
**Total (95% CI)**		**1156**		**3211**	**100.0%**	**1.92 [0.57, 6.51]**
Total events	881		2308			

Heterogeneity: $Tau^2 = 1.86$; $Chi^2 = 115.59$, df = 4 (P < 0.00001); $I^2 = 97\%$
Test for overall effect: Z = 1.05 (P = 0.29)

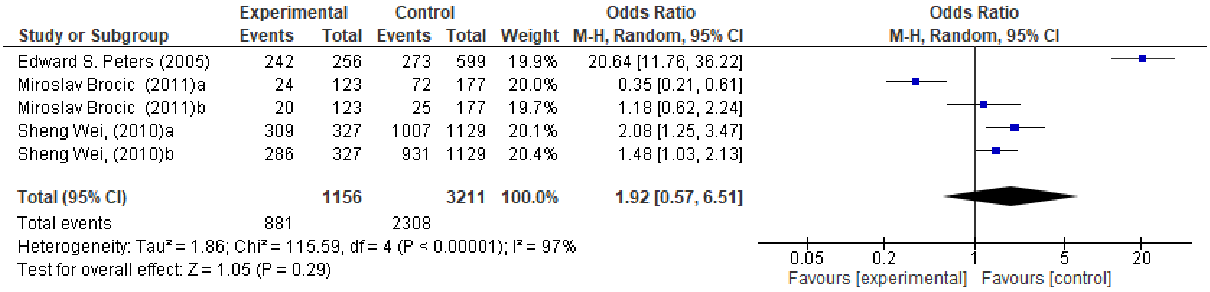

Supplementary Figure 29: Assessment of strength of association between family history and gene affected

	Cases		Control			Odds Ratio
Study or Subgroup	Events	Total	Events	Total	Weight	M-H, Random, 95% CI
C. Yapijakis 2007	54	144	0	153	8.1%	184.88 [11.28, 3029.89]
Eleftherios Vairaktaris 2006	58	160	65	153	15.7%	0.77 [0.49, 1.21]
Hsin-I Huang MS 2018	85	282	98	324	15.8%	1.00 [0.70, 1.41]
Li-Wang 1998	28	60	58	112	15.3%	0.81 [0.43, 1.53]
Paola Gallì 2009	84	283	53	295	15.8%	1.93 [1.30, 2.85]
R-R Rosa 2021	10	28	28	45	14.3%	0.34 [0.13, 0.90]
Sarika Daripall 2015	7	350	324	535	15.0%	0.01 [0.01, 0.03]
**Total (95% CI)**		**1307**		**1617**	**100.0%**	**0.70 [0.23, 2.18]**
Total events	326		626			

Heterogeneity: $Tau^2 = 2.06$; $Chi^2 = 158.61$, df = 6 ($P < 0.00001$); $I^2 = 96\%$

Test for overall effect: Z = 0.61 (P = 0.54)

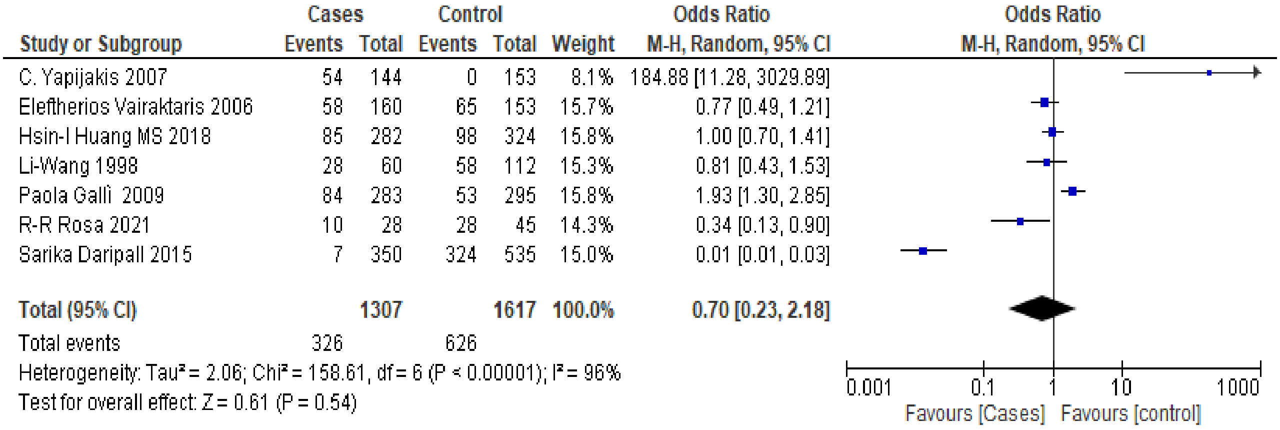

Supplementary Figure 30: Assessment of strength of association between chronic mucosal trauma and gene affected

Study or Subgroup	Cases Events	Cases Total	Control Events	Control Total	Weight	Odds Ratio M-H, Random, 95% CI
Canan Cacina 2018	33	114	66	165	50.0%	0.61 [0.37, 1.02]
Hakan Avci 2018	65	111	46	148	50.0%	3.13 [1.87, 5.24]
**Total (95% CI)**		**225**		**313**	**100.0%**	**1.38 [0.28, 6.87]**
Total events	98		112			

Heterogeneity: $Tau^2 = 1.27$; $Chi^2 = 19.57$, df = 1 ($P < 0.00001$); $I^2 = 95\%$

Test for overall effect: Z = 0.40 (P = 0.69)

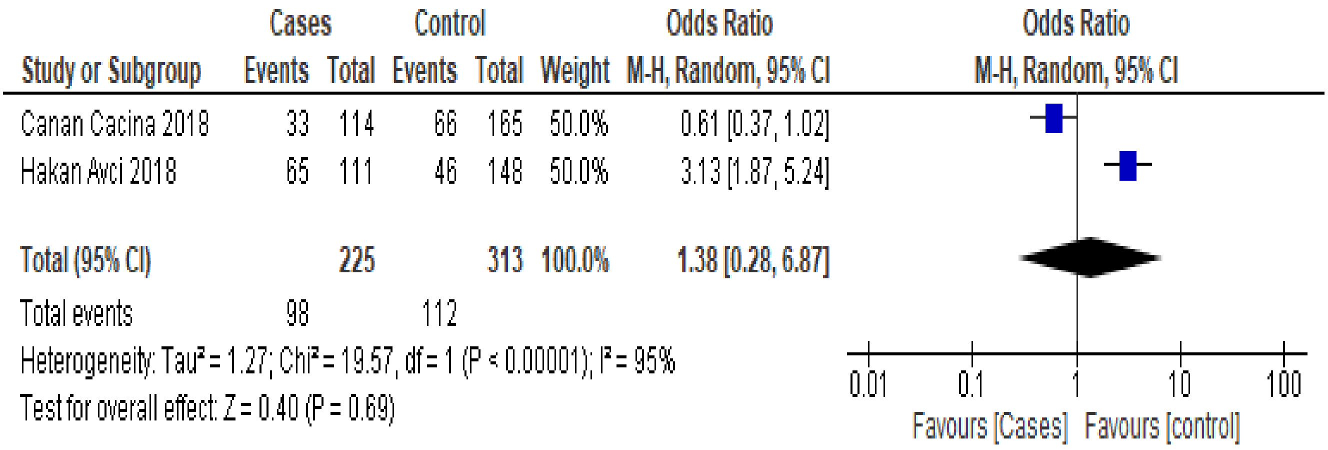

Supplement: Supplementary file 2 — Supplementary Information [file 43856_2026_1398_MOESM2_ESM.pdf]
