# Supplementary material for: Assessment of genetic susceptibility in patients with oral squamous cell carcinoma: a systematic review and meta-analysis
Source: Commun Med (Lond). 2026 Mar 20;6:406. doi: 10.1038/s43856-026-01398-9 (PMC13396505; doi:10.1038/s43856-026-01398-9)
Supplement: Supplementary file 3 — Description of Additional Supplementary files [file 43856_2026_1398_MOESM3_ESM.pdf]

Description of Additional Supplementary Files

Supplementary Data 1-Data Sheet showing extracted data of included studies

Supplementary Data 2- Data sheet for meta-analysis

Supplementary Data 3- Data sheet for subgroup analysis of environmental risk factors tobacco and alcohol for genetic susceptibility of development of oral squamous cell carcinoma

Supplementary Data 4- Data sheet for assessment of risk of bias assessment for included studies
