# Supplementary material for: Assessment of genetic susceptibility in patients with oral squamous cell carcinoma: a systematic review and meta-analysis
Source: Commun Med (Lond). 2026 Mar 20;6:406. doi: 10.1038/s43856-026-01398-9 (PMC13396505; doi:10.1038/s43856-026-01398-9)
Supplement: Supplementary file 1 — Transparent Peer Review file [file 43856_2026_1398_MOESM1_ESM.pdf]

nature portfolio

Peer Review File

Assessment of genetic susceptibility in patients with Oral squamous cell carcinoma: a systematic review and meta-analysis.

Corresponding Author: Dr KIRAN Jadhav

This file contains all reviewer reports in order by version, followed by all author rebuttals in order by version.

Version 0:

Reviewer comments:

Reviewer #1

(Remarks to the Author)
A very extensive work written beautifully. But the author could include forest plot displaying the meta analysis which will help the readers to understand better about the topic especially for the random effect model.

Reviewer #2

(Remarks to the Author)
The authors have made an appreciable effort to answer the question why only some patients are more susceptible to oral cancer compared to others although they have similar history of tobacco and/ alcohol abuse. The true association of genetic polymorphisms with oral cancer risk can be identified by including patients with oral cancer without a history of substance abuse (like chronic trauma as an etiology). Comparing the pattern of gene polymorphisms in both the groups may help to solve this enigma.

Version 1:

Reviewer comments:

Reviewer #2

(Remarks to the Author)

Open Access This Peer Review File is licensed under a Creative Commons Attribution 4.0 International License, which permits use, sharing, adaptation, distribution and reproduction in any medium or format, as long as you give appropriate credit to the original author(s) and the source, provide a link to the Creative Commons license, and indicate if changes were made.
In cases where reviewers are anonymous, credit should be given to 'Anonymous Referee' and the source.
The images or other third party material in this Peer Review File are included in the article's Creative Commons license, unless indicated otherwise in a credit line to the material. If material is not included in the article's Creative Commons license and your intended use is not permitted by statutory regulation or exceeds the permitted use, you will need to obtain permission directly from the copyright holder.
To view a copy of this license, visit https://creativecommons.org/licenses/by/4.0/

Sr. No.	Reviewers comments	Reply by authors	Correction done page and line no.
1	Author could include forest plot displaying the meta analysis which will help the readers to understand better about the topic especially for the random effect model.	Dear Reviewer thank you for your valuable suggestion for improvement of our manuscript. We had already uploaded many forest plots as supplementary material. As per suggestions we have now included some specific forest plots of random effect model analysis and subgroup analysis in main manuscript as images.	Figure 3 to Figure 9 are included in manuscript file
2	The true association of genetic polymorphisms with oral cancer risk can be identified by including patients with oral cancer without a history of substance abuse (like chronic trauma as an etiology) Comparing the pattern of gene polymorphisms in both the groups may help to solve this enigma.	Dear reviewer we really appreciated your suggestion to extend the analysis to include non substance associated oral squamous cell carcinoma. We could extract and analyze the data related to family history and chronic mucosal trauma as non substance factor for Oral squamous cell analysis	Line 312-327 and 406 to 426,<br>Check for supplementary file S6<br>Three references 201, 202 and 203 are added to support the literature.
